# Non-invasive screening in hereditary cancer: a randomized controlled trial to test cell-free DNA-based early detection in the CHARM consortium

**DOI:** 10.1038/s41431-026-02014-z

**Published:** 2026-01-29

**Authors:** Kirsten M. Farncombe, Julia A. Sobotka, Melyssa Aronson, Mark Basik, Yvonne Bombard, Leslie Born, Rona Cheifetz, Marc Clausen, Natalie Coburn, Lesa Dawson, Andrea S. Doria, Khaled Y. Elbanna, Holly Etchegary, William D. Foulkes, Chiquita Hessels, Angela Hyde, Karineh Kazazian, Adam Kinnaird, C. Anne Koch, Stephane Laframboise, Jordan Lerner-Ellis, Stephanie Lheureux, David Malkin, Ur Metser, Lynette S. Penney, Sarah Ridd, Kasmintan A. Schrader, Teresa Tiano, Alicia A. Tone, Patrick Veit-Haibach, Stephanie Wong, Wei Xu, Trevor J. Pugh, Raymond H. Kim

**Affiliations:** 1 Princess Margaret Cancer Centre, University Health Network, Toronto, ON Canada; 2 Zane Cohen Centre for Digestive Diseases, Sinai Health, Toronto, ON Canada; 3 Department of Surgery, McGill University Medical School, Montreal, QC Canada; 4 Department of Oncology, McGill University Medical School, Montreal, QC Canada; 5 Institute of Health Policy, Management and Evaluation, University of Toronto, Toronto, ON Canada; 6 Genomics Health Services Research Program, Li Ka Shing Knowledge Institute, St. Michael’s Hospital, Unity Health Toronto, Toronto, ON Canada; 7 BC Cancer, Vancouver, BC Canada; 8 ICES, Toronto, ON Canada; 9 Odette Cancer Centre, Sunnybrook Health Sciences Centre, Toronto, ON Canada; 10 Department of Surgery, Sunnybrook Health Sciences Center, Toronto, ON Canada; 11 Department of Obstetrics and Gynecology, University of British Columbia, Vancouver, BC Canada; 12 Department of Diagnostic & Interventional Radiology, Research Institute, The Hospital for Sick Children, Toronto, ON Canada; 13 Department of Medical Imaging, University of Toronto, Toronto, ON Canada; 14 NLSUPPORT, Memorial University of Newfoundland, St. John’s, NL Canada; 15 Faculty of Medicine, Memorial University of Newfoundland, St. John’s, NL Canada; 16 Department of Human Genetics, McGill University, Montreal, QC Canada; 17 Dr. H. Bliss Murphy Cancer Center, St. John’s, NL Canada; 18 Department of Surgery, University of Toronto, Toronto, ON Canada; 19 Department of Surgical Oncology, Toronto General Hospital, University Health Network, Toronto, ON Canada; 20 Department of Surgery, University of Alberta, Edmonton, AB Canada; 21 Radiation Medicine Program, Princess Margaret Cancer Centre, University Health Network, Toronto, ON Canada; 22 Department of Radiation Oncology, University of Toronto, Toronto, ON Canada; 23 Department of Obstetrics and Gynecology, University of Toronto, Toronto, ON Canada; 24 Division of Gynecology Oncology, University Health Network, Sinai Health System, Toronto, ON Canada; 25 Laboratory Medicine and Pathobiology, University of Toronto, Toronto, ON Canada; 26 Lunenfeld-Tanenbaum Research Institute, Sinai Health System, Toronto, ON Canada; 27 Pathology and Laboratory Medicine, Mount Sinai Hospital, Sinai Health System, Toronto, ON Canada; 28 Division of Medical Oncology and Hematology, Princess Margaret Cancer Centre, University Health Network/Sinai Health System, Toronto, ON Canada; 29 Department of Medical Oncology and Hematology, University of Toronto, Toronto, ON Canada; 30 Division of Hematology-Oncology, Hospital for Sick Children, Toronto, ON Canada; 31 Department of Pediatrics, University of Toronto, Toronto, ON Canada; 32 Department of Medical Biophysics, University of Toronto, Toronto, ON Canada; 33 University Medical Imaging Toronto, Joint Department of Medical Imaging, University Health Network, Mount Sinai Hospital, Women’s College Hospital, Toronto, ON Canada; 34 Dalhousie University, Halifax, NS Canada; 35 University of British Columbia, Vancouver, BC Canada; 36 Ovarian Cancer Canada, Toronto, ON Canada; 37 Ontario Institute for Cancer Research, Toronto, ON Canada; 38 The Sir Mortimer B. Davis Jewish General Hospital, Montreal, QC Canada; 39 Division of Clinical and Metabolic Genetics, The Hospital for Sick Children, Toronto, ON Canada; 40 Department of Medicine, University of Toronto, Toronto, ON Canada

**Keywords:** Cancer genetics, Cancer prevention

## Abstract

Individuals with hereditary cancer syndromes are born with germline genetic variants that significantly increase their lifetime risk of developing multiple cancers. Cancer rates and overall mortality can be reduced with intensive surveillance to facilitate early cancer detection. However, participating in diagnostic imaging and endoscopy surveillance programs is often time-consuming, overwhelming, inconvenient, and anxiety-inducing. To improve this, multi-cancer early detection tests are being developed using cell-free DNA (cfDNA) sequencing analysis to detect cancers with more sensitivity than conventional screening methods. Our community (the CHARM consortium: Cell-free DNA in Hereditary And high-Risk Malignancies) has been exploring the use of cfDNA sequencing in hereditary cancer, and has launched the CHARM2 prospective randomized controlled trial, which is enrolling 1000 participants with Hereditary Breast and Ovarian Cancer, Lynch syndrome, Li-Fraumeni syndrome, Neurofibromatosis type 1 and Hereditary Diffuse Gastric Cancer to improve equitable access, early detection and surveillance for high-risk individuals. All participants will have screening as per conventional syndrome-specific surveillance recommendations. Half the participants (experimental cohort) will also have cfDNA analysis at least three times a year, with abnormal results triggering dedicated clinical imaging and diagnostic evaluation, and heightened surveillance. Vetted by our patient advisors, validated patient-reported outcome and experience measures assessing participant psychosocial outcomes, engagement, and test preferences will be administered to both arms. Our goal is to inform if and how cfDNA analysis could be implemented into routine clinical care and offer a path to equitable and more convenient cancer screening for all high-risk Canadians.

## Hereditary cancer syndromes and surveillance

Individuals with a hereditary cancer syndrome (HCS) have an inherited pathogenic or likely pathogenic variant (P/LP) in one of the >100 genes contributing to these conditions, and are at an increased risk of developing multiple types of cancers [1]. Some HCS responsible for high fatality cancers are: Hereditary Breast and Ovarian Cancer (HBOC; *BRCA1*, *BRCA2* and *PALB2*)—increased risk of developing female and male breast, ovarian, prostate, and pancreatic cancers [2, 3], Lynch syndrome (LS; *MLH1, MSH2/EPCAM, MSH6* and *PMS2*)—increased risk of developing colorectal, endometrial, ovarian, gastric, urinary tract, biliary tract, brain, prostate and skin cancers, with the specific variant affecting the risk of each type of cancer [4], Li-Fraumeni syndrome (LFS; *TP53*)—at risk of developing almost all types of cancers, most commonly soft tissue sarcomas, osteosarcomas, breast cancer, brain tumors, adrenocortical carcinomas, leukemias and lung cancer [5], Neurofibromatosis type 1 (NF1; *NF1*)—develop benign and malignant multisystem manifestations and are at risk of central and peripheral nervous system and breast cancers, as well as gastrointestinal stromal tumors and pheochromocytomas; transformation of benign plexiform neurofibromas into the highly aggressive malignant peripheral nerve sheath tumor is a leading cause of early death in this population [6], and Diffuse Gastric and Lobular Breast Cancer syndrome (DGLBCS; *CDH1*)—at a high risk for developing diffuse gastric cancer and lobular breast cancer [7].

There are other recessive conditions associated with extremely high risks of developing early-onset cancer. This includes childhood predisposition syndromes: constitutional MMR deficiency (CMMRD), associated with a family history of LS and caused by biallelic pathogenic variants in LS genes that leads to an increased risk of brain cancer, leukemia and gastrointestinal tumors [8], as well as recessive Fanconi anemia, caused by biallelic *BRCA2* and *PALB2* pathogenic variants and leads to an increased risk of developing leukemia, head and neck cancers, and gynecologic cancers [9].

Once a germline P/LP variant is identified, individuals begin lifelong surveillance protocols, tailored to their specific HCS and under the care of multiple medical specialists, with the aim of identifying and treating cancer at an early stage [4, 10]. These individuals undergo a continuous lifecycle of previvor to cancer patient to cancer survivor (Supplementary Fig. S1). Common surveillance strategies include diagnostic imaging (e.g., MRI, mammography, ultrasound), tumor markers (e.g., CA125, prostate-specific antigen) and cystoscopy/endoscopy (e.g., esophagogastroduodenoscopy, colonoscopy). For certain cancer types (i.e., pancreas, ovaries), there are no proven methods of effective early detection. As a result, many individuals will choose to undergo cancer risk-reducing surgeries (i.e., mastectomy, salpingo-oophorectomy ± hysterectomy) or have no alternatives, resulting in a high degree of cancer anxiety [11, 12].

## Limitations of current surveillance protocols

Individuals with HCS undergo highly specific surveillance on at least an annual basis, with increased frequency for HCS associated with childhood-onset cancers. These surveillance programs are highly reliant on centralized clinical expertise and often require traveling a great distance for access to specialized, costly equipment [13]. Those living in rural or remote regions often face inequitable access to the specialized genetics and oncology clinics that are typically only available in more urban locations.

The techniques and general surveillance process have been described by individuals as unpleasant, burdensome, overwhelming, exhausting, time-consuming, costly and insufficient [14–16]. Individuals may also face “scanxiety”, which is increased anxiety and/or distress related to cancer tests, including MRIs and other diagnostic imaging, before the process and until the results are disclosed [17]. Reviews on scanxiety have been conducted [18–20], including research into patients with advanced cancer [21, 22], and cancer survivors [23–26]. Furthermore, for individuals who choose to undergo life-altering surgeries, the reduction in cancer risk from preventive surgery will be accompanied by significant psychological and physical sequalae.

## Development of the CHARM consortium

The CHARM consortium (cfDNA in Hereditary And High-Risk Malignancies) was founded in 2017 to address hereditary cancer care and delivery gaps, including surveillance and early cancer detection through the evaluation of blood-based cfDNA testing [27]. To account for the geographic distribution across Canada, 11 sites encompassing 9 provinces/territories were engaged. CHARM focuses on the enrollment of adults and children with more common HCS—HBOC, LFS, NF1 and LS, with participants providing written informed consent. The CHARM consortium includes an agreement that governs the intellectual property (IP) and secondary use of data and samples generated from all past and future CHARM studies. Participating sites have both individual clinical trial agreements for funding and center-specific research ethics board policies, with the lead bio-/data-bank (University Health Network—UHN), and a master participation agreement (consortium agreement) that includes the sharing, use, and governance of data generated retrospectively, ongoing or in the future from any CHARM-related study. Participating sites maintain exclusive IP rights to data provided to the consortium, and have agreed to core data elements, data definitions and ontologies. The consortium’s mission is: (1) to establish a nation-wide plasma biobank and data repository for HCS patients, (2) advance circulating-tumor DNA (ctDNA) testing development, and (3) optimize integration of ctDNA into clinical practice. The database currently contains approximately 2400 participants, and will continue to expand with additional CHARM trials and as more centers join the consortium. Each of the participating sites provides clinical data from electronic health record systems, and data, including longitudinal plasma samples, tumor tissue, genomic and epigenomic data, clinical data, and participant preferences and health services, which are harmonized and deidentified prior to analysis. Deidentified data is available to all members of the CHARM consortium, with standardized clinical data abstraction following Marathon of Hope Cancer Centers Network standards [28], and genome-wide fragment data available through the Phenomic Liquid Biopsy Resource. The consortium agreement adheres to each collaborating institution’s respective privacy and data protection laws, including Canada’s overarching Personal Information Protection and Electronic Documents Act.

## The advancement of cell-free DNA and its application in hereditary cancer

To address the ongoing limitations of HCS surveillance, a shift has been made towards an accessible approach that utilizes a single, highly quantitative platform that is effective across all types of hereditary cancer—cell-free DNA (cfDNA) analysis. CfDNA analysis in oncology—also known as liquid biopsy—relies on the identification and analysis of ctDNA fragments released into the blood plasma [29]. Multiple approaches are being trialed, including whole genome sequencing (WGS), transcriptome sequencing, fragmentomics, methylation (i.e., cell-free methylated DNA immunoprecipitation and high-throughput sequencing—cfMeDIP-seq), structural variants (SV), and T-cell receptor (TCR) repertoire analysis. Results from recent studies have illustrated a connection between epigenetic changes and cfDNA fragmentation [30], with changes resulting from genomic, epigenomic, transcriptomic and chromatin states impacting size, position, coverage, mutation, structural and methylation characteristics of cfDNA [31]. The ability to identify and track these changes may improve disease identification, including early cancer detection [30, 31]. Plasma cfDNA methylation profiling has shown different methylation profiles between individuals diagnosed with *BRCA1/2* and breast cancer, compared to healthy controls without a germline *BRCA1/2* pathogenic variant [32]. Similarly, an SV-based ctDNA assay could detect ctDNA in 96% of participants at baseline with early-stage breast cancer, illustrating its potential clinical utility [33]. Finally, TCR (expressed by T cell lymphocytes) within cfDNA can provide information about how T cells respond to adaptive immune responses, including infectious pathogens or immunotherapies [34, 35]. These modalities all support the use of cfDNA in detecting cancer early, particularly in individuals with hereditary cancer.

## Evolution of liquid biopsies and the multi-cancer early detection test

Recently, cfDNA analysis has been used in multi-cancer early detection (MCED) tests, where cfDNA analysis is performed on blood samples to identify the presence and origin of cancer. This includes the CancerSEEK test which used next generation sequencing of genetic mutations and protein biomarker levels in plasma, in combination with diagnostic PET-CT to detect breast, colorectal, esophageal, liver, lung, ovarian, pancreatic and stomach cancers (DETECT-A study) [36], the OverC test, based on cfDNA methylation analysis, which demonstrated high sensitivity, specificity and accuracy of cancer signal origin (CSO—most likely tumor type) in colorectal, esophageal, liver, lung, ovarian and pancreatic cancers (THUNDER study; NCT04820868) [37], the EpiPanGI Dx test that used cfDNA methylation for the early detection of gastrointestinal cancers [38], and the Geneseeq MERCURY test on plasma cfDNA using genetic- and fragmentomics-based features derived from low-coverage WGS developed and validated for breast, cervical, colorectal, endometrial, esophageal, gastric, liver, lung, ovarian, pancreatic, prostate, bile duct and lymphoma (NCT06011694) [39].

GRAIL LLC has conducted some of the most extensive and largest trials in MCED testing with the development of the Galleri MCED test, based on methylation patterns of cfDNA and machine learning classifiers to detect >50 individual cancer types and predict CSO, which was developed, refined and validated by the Circulating Cell-Free Genome Atlas study (CCGA; NCT02889978) [40, 41]. The Galleri test detected cancers that lack screening tests (i.e., bile duct, small intestine, pancreas, spindle cell neoplasm, and hematologic malignancies), and had a < 1% false positive rate [42]. WG methylation was determined to be the most sensitive method, best predicted CSO and should be used for a targeted methylation MCED test [43]. However, the detection rate varies by cancer type, with a lower sensitivity for breast cancer in asymptomatic populations [44]. MCED tests were used in the PATHFINDER study (NCT04241796) [42, 45], the UK National Health Service (NHS) randomized control trial (ISRCTN 91431511; NCT05611632) [46], and, most recently, the PATHFINDER 2 study (NCT05155605) (follow-up analysis ongoing).

MCED blood tests show potential benefits of the ability to screen for many cancers, reduced invasiveness compared to current screen modalities and procedural familiarity; however, there are still concerns about false-negative and false-positive results and increased anxiety [47, 48]. Barriers to MCED acceptance include psychological concerns, limited knowledge about cancer screening, trust in health care systems and provider relationships, and accessibility [49]. To advance clinical care and equity through the adoption of MCED tests, trials must show direct benefit in reducing cancer-specific mortality and undergo independent evaluation [50].

## Results from the CHARM consortium

The CHARM consortium has published a comprehensive review of four common HCS (HBOC, LS, LFS, NF1), including surveillance strategies, the transition to liquid biopsy in hereditary cancer early detection and preliminary findings [27]. We have also published two qualitative interpretative descriptive studies exploring Canadian patient and provider preferences regarding the utility of liquid biopsy testing for early cancer detection in HCS; although enthusiasm was expressed from both participants and providers, they also noted concerns, including false positive/negative results, additional anxiety and further investigations downstream, increasing invasiveness [51, 52]. CfDNA sequencing and analysis have been published for the LFS cohort, where a multi-modal liquid biopsy assay (targeted gene panel, shallow whole genome sequencing (sWGS) and cfMeDip-seq) approach increased the detection rate in participants with an active cancer diagnosis and could identify cancer-associated signal(s) before conventional surveillance strategies [53].

Current sequencing analysis for CHARM is investigating HBOC, NF1, and LS data. Similar to the LFS cohort, in the HBOC cohort, a multi-modal liquid biopsy assay integrating genomic, fragmentomic and methylation features detected cancer-associated signals in 73.8% of *BRCA1/2* individuals with cancer, supporting the potential of longitudinal, multi-modal analysis in HBOC early detection [54]. In the NF1 cohort, initial results indicate that cfDNA analyses can differentiate between patients classified by the clinical care team (medical geneticists, neurologists, genetic counselors) as high- or low-severity NF1, based on clinical presentation, through nucleosome positioning data [55].

In this paper, we describe the protocol for the CHARM2 randomized controlled trial, which is enrolling 1000 participants with five hereditary cancer syndromes into balanced control and experimental arms. In addition, we highlight results to date for participants receiving a cancer signal detected and follow-up workflows.

## Expansion to the CHARM2 randomized controlled trial

In 2023, we expanded the CHARM consortium to conduct the first trial of its kind in Canada (CHARM2) to: (1) compare cancer detection yields using cfDNA testing at least three times a year vs current standard of care surveillance protocols, (2) assess whether cfDNA testing detects cancer earlier, increases access to medical treatment and decreases morbidity and mortality, and (3) understand the patient experience and outcomes with cfDNA-imaging testing.

One thousand participants are being enrolled from nine sites across Canada: University of Alberta (Alberta), BC Cancer (British Columbia), IWK Health (Nova Scotia), Jewish General Hospital (Quebec), NL Health Services (Newfoundland and Labrador), the Hospital for Sick Children, Sinai Health System, Women’s College Hospital and UHN (Ontario) (Supplementary Fig. S2). Enrolled participants are heterozygous for a P/LP variant in one of the following HCS: HBOC (*BRCA1, BRCA2* and *PALB2*), LS (*MLH1, MSH2, EPCAM, MSH6* and *PMS2*), DGLBCS (*CDH1*), NF1 (*NF1*) and LFS (*TP53*). Participants who harbor a variant of uncertain significance (VUS) that is being managed clinically as pathogenic based on variant evidence, and family and personal history, may also be enrolled.

### Eligibility criteria

To be eligible for enrollment, participants must have completed their cancer treatment a minimum of 3 years from the time of enrollment, as the aim of our study is early detection of a new primary cancer or a recurrence. Patients on maintenance PARP therapy (i.e., olaparib) are able to enroll if there has been no recurrence within three years. Similar to other MCED trials [39, 45], participants with non-invasive squamous cell carcinoma, basal-cell carcinoma, ductal carcinoma in situ, serous tubal intraepithelial carcinoma, and stromal tumor infiltrating lymphocytes diagnosed within three years from the time of enrollment are eligible. Participants with LFS can be enrolled at any age; for other cohorts (HBOC, LS, DGLBCS and NF1), individuals must be 18 years of age or older. There is no upper age limit. Participants who have had prophylactic surgery are still eligible (i.e., a *BRCA* heterozygote who has had a mastectomy and bilateral salpingo-oophorectomy), as these individuals may develop other cancers unrelated to the prior surgical procedure (i.e., pancreas) and there may be a small residual risk post-surgery. Individuals who have not yet started their surveillance protocols (i.e., a *BRCA* heterozygote who is in her early twenties before the age of recommended breast surveillance) and pregnant women are also eligible. We plan to conduct subgroup analysis of each of these groups, stratifying for HCS and including those with risk reduction surgeries. We acknowledge that groups with risk-reduced *BRCA1/2* may have significantly decreased cancer risks; however, their inclusion also allows for data capture of rare outcomes through the longitudinal assessment of potential non-breast/ovarian cancer events. In particular, given that pancreatic screening is not uniform in Canada, pancreas cancer is a prominent concern for many *BRCA1/2* heterozygotes [56]. The only exclusion criterion is that individuals with *CDH1* need to have stomachs and/or breasts intact, as there is limited evidence in the literature suggesting residual risks for other cancers. In contrast, given that the residual risk of subsequent cancers in *CDH1* heterozygotes was exceedingly low, these patients were excluded from the study [57, 58].

### Randomized controlled trial protocol

**Fig. 1:**
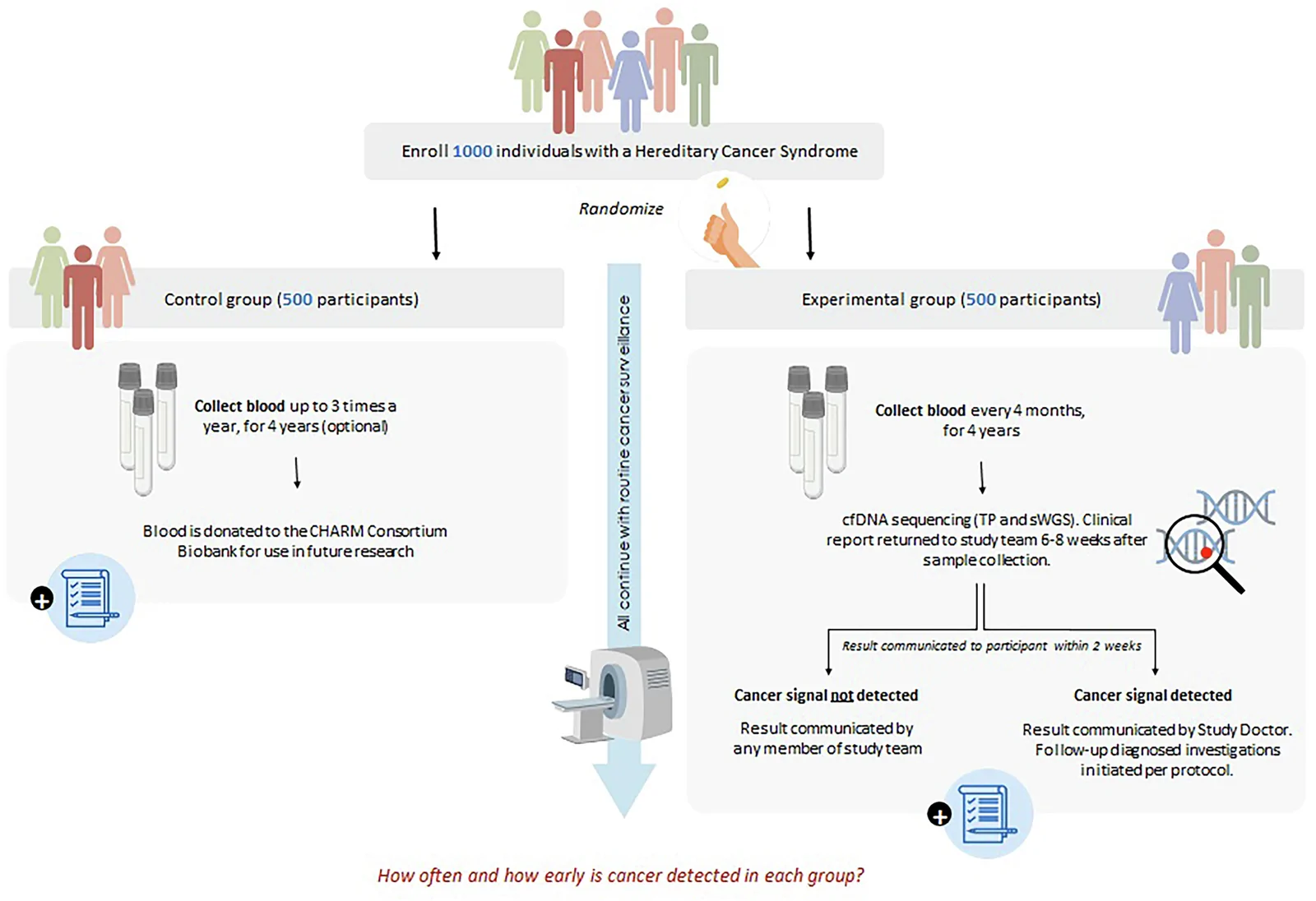
CHARM2 workflow. Randomized controlled trial protocol illustrating the recruitment process for control and experimental groups.

An overview of the protocol is summarized in Fig. 1. Participants are recruited from a variety of sources, including genetics/oncology clinics and high-risk screening programs across Canada, and are enrolled into the randomized controlled trial, with the experimental group undergoing cfDNA sequencing every 4 months. Power analysis is based on the primary outcome of improved cancer detection at various diagnosis rate scenarios of the study cohort at the end of the study, range with current 5% detection rate for all HCS without cfDNA modeling, up to 10% should cfDNA improve detection. Based on the power analysis, our study with 1000 patients will achieve >97% statistical power to detect at least 2% increased diagnosis rate attributable to cfDNA testing with a 2-sided significance level at 0.05.

The only variable controlled for during randomization is the cancer syndrome (50% experimental participants/50% control participants). Half of the participants will undergo their regular standard of care surveillance (control group), whereas the other half will also have cfDNA analysis alongside their standard of care screening (experimental group). For abnormal cfDNA findings, the study physician will then order additional testing (combination of imaging, scoping, physical examination and bloodwork, as appropriate). This trial collects data that can be used to compare the rate of cancer detection using cfDNA-informed surveillance (experimental group) with the rate of detection in the arm that did not receive the result of the cfDNA test but was managed according to current conventional screening programs (control group). Control group participants have the option to donate blood samples (0–3 samples per year) to the CHARM Biobank over the duration of the trial. These biobank samples may be used in future research studies, for example, as a validation cohort.

Experimental group participants provide a blood sample (30 mL, 3 Streck tubes) every 4 months (three times/year) for four years, starting from the time of their enrollment. In Newfoundland and Labrador, Ontario and British Columbia, blood can be collected at the hospital or at a community laboratory. To date, 75% of Ontario samples have been collected at a community laboratory, which has improved access to the trial for participants living in more rural regions. At sites where blood work is only collected at the hospital, the study provides compensation for travel over 40 km. Following blood collection, samples are shipped weekly from each site to the Ontario Institute for Cancer Research (OICR) Genomics Program in Toronto, Ontario, for cfDNA testing. The cfDNA blood samples are stable at room temperature for up to 14 days after collection. A repeat blood collection is performed if not enough cfDNA is extracted to run the test. OICR Genomics offers College of American Pathologists (CAP)/Accreditation Canada Diagnostics (ACD), Clinical Laboratory Improvement Amendments (CLIA)-certified, International Organization for Standardization (ISO) 15189-compliant services for clinical reporting and research requirements. Results are available approximately 6-8 weeks after sample collection (51 days, on average, with a minimum of 35 and a maximum of 78 days, not accounting for redraws). A clinical report is generated for all results, with negative results disclosed to the participant (by phone or email, depending on participant preference) by the study team research coordinators, and positive results disclosed by the clinical lead or study investigator of the respective study site by phone.

Blood samples undergo two methods of sequencing at OICR: (1) a 10,000x targeted gene panel to detect somatic mutations in the cfDNA (400X deduplicated coverage with clinically-reportable limit of detection of 1%), and (2) sWGS to detect copy number changes across the genome (clinically-reportable limit of detection of 10%). The targeted gene panel includes 14 genes associated with hereditary cancer (*BRCA1, BRCA2, PALB2, TP53, APC, EPCAM, PMS2, MLH1, MSH2, MSH6, CCNE1, CDH1, VHL* and *NF1*). All introns and exons are sequenced for *BRCA1/2*, and all exons are sequenced for the other genes included on the panel. The sWGS method uses the ichorCNA algorithm to quantify the copy number changes and predict tumor burden. A positive result for either panel (somatic mutation at a VAF > 1%) or sWGS (tumor burden >10%) will trigger follow-up investigations by the clinical study team. A positive sWGS or panel result is not tissue-specific. Reflex methylation testing (enzymatic methyl-seq) on the remaining sample is initiated following a positive result with the goal of building a database and developing methylation signatures to enable tissue of origin prediction for less-prevalent cancers.

### Surveillance work-up for positive results

The cfDNA surveillance follow-up protocols for positive results (Supplementary Figs. S3–S18) were developed by a multidisciplinary working group of 15 clinicians, including radiologists, gynecologic, medical, pediatric and surgical oncologists, geneticists and genetic counselors. Our positive study blood test result is a pan-cancer positive signal (i.e., it does not identify a tissue-specific organ) and is not guided by methylation testing; follow-up investigation is guided by the participant’s P/LP variant, rather than targeted towards the general population. As a result, the follow-up protocols consider the participant’s gene variant, surgical history (i.e., risk-reducing surgeries), cancer history, and sex assigned at birth. Although diagnostic workups are proposed, the exact procedures follow the clinician’s judgment and are tailored to the participant’s risk factors and personal preferences. If a participant has completed one of the follow-up investigations as part of their clinical care recently, the clinical lead for the respective site will decide if the investigation was sufficient or if the test should be repeated. The order of the follow-up investigations considers the likelihood of a given cancer and takes into consideration the associated cost, important information to gather to guide future implementation in a public health care system. For example, as tumor markers are inexpensive tests, a work-up for all tumor markers is ordered at the beginning of the workflow. The protocols include a combination of a physical exam, blood work and imaging, with diagnostic workflow results from other MCED studies indicating that an imaging strategy (e.g., PET-CT) is an effective test for resolving a positive MCED test [59].

As the follow-up surveillance protocols are triggered by a research result, the tests are funded through research, which is conducted at a frequency and for a reason that is above the standard of care (e.g., if a participant normally has a yearly breast MRI but their cfDNA test comes back as positive, they would be able to have an interval breast MRI). This reduces the typical wait times encountered with clinical testing in a public health care setting, as many institutions have separate time slots available for research studies, which decreases imaging wait time.

### Participant experience and engagement outcomes

To understand and compare the patient experience before, during and after the cfDNA trial versus control arms, an observational mixed methods study will be conducted using qualitative interviews and validated measures, following guidance for reporting qualitative (COREQ) [60] and observational studies (STROBE) [61]. The NHS-Galleri trial followed a similar assessment, where participants with a cancer signal detected received questionnaires at three timepoints to assess anxiety using the short-form 6-item Spielberger State Trait Anxiety Inventory, and psychological consequences of screening using the Psychological Consequences of Screening Questionnaire, as well as other behaviors [62]. The CHARM2 trial will test whether a programmatic approach to cfDNA testing decreases distress, uncertainty and anxiety, and increases quality of life and patient empowerment using the validated Hospital Anxiety and Depression Scale (HADS) [63]. We acknowledge that these participants are a high-risk group that experiences scanxiety and may still have increased anxiety while waiting for the results of their blood test. We anticipate that the more frequent screening from imaging and accessible blood tests versus just imaging may reduce overall anxiety, as participants have more data/information in the interim and will see changes more regularly. We are assessing the impact of cfDNA screening at least three times a year using validated scales, prioritized by our patient advisors [64–66], and quality of life measured by the 12-item Short Form Survey (SF-12) [67]. The questionnaires also include questions around satisfaction with the study procedures (i.e., frequency of blood collections of the study, result disclosure process) for experimental participants. This will help guide future clinical implementation of cfDNA surveillance. To mitigate potential biases of participants’ questionnaire responses being impacted by knowing their cohort, baseline questionnaires were taken from all participants prior to randomization. The trial was designed in collaboration with patient partners, who emphasized the importance of participants knowing their cohort.

All questionnaires are completed electronically through REDCap. Prior to the initiation of the trial, four patient advisors were enlisted to join the patient advisory group and helped codevelop the study design, procedures, and select relevant and appropriate patient-centered measures and interview questions. Control group participants receive the link to the questionnaire every four months from the date of their enrollment. Experimental group participants receive a link to the questionnaire following the disclosure of each study blood test result (approximately every four months). Completion of questionnaires three times a year continues until the end of the trial. If a participant is diagnosed with cancer, they no longer complete questionnaires.

## CHARM2 results to date

To date, we have enrolled 575 participants (322 HBOC, 205 LS, 27 LFS, seven DGLBCS, and 14 NF1). Distribution per HCS is due to historic reasons, where certain clinics (e.g., BRCA1/2 and LS) have been well-established for decades, whereas other clinics are more recently established (e.g., NF1). We have collected 474 samples from the experimental group participants, with sequencing in progress for 115 participants, and have completed, with results returned for 359 participants. 82 participants have provided a single sample, 44 participants have provided two samples, 76 participants have provided three samples, and 20 participants have provided four samples. The average time between serial samples has been 129 days (4.2 months).

The average time from date of sample collection to date of result disclosure is 54 days. At the time of result disclosure, the study team obtains a medical history update from the participant, including the results of any interim routine cancer screening test. 397 negative results have been returned; of these, 395 are true negatives (i.e., patient does not have cancer based on routine screening) and two were false negatives. Three positive results have been returned, with a confirmed tumor found for two participants, and investigations are in progress for the third participant. Thus far, the questionnaire completion rate for the control participants is 64%, and 71% for the experimental participants. There are no interim results as the analysis of the questionnaires will be performed once the trial is completed. Likewise, no cancer detection results from the control group have been analyzed so far, to preserve the blinded, randomized nature of the experimental design until formal comparison at the completion of the study.

### Positive case 1

#### Demographics and clinical background

A female in her late 60 s with a germline *BRCA1* variant in c. 185delAG; p.Glu23fs*17 (also commonly known as c.68_69del; p.Glu23fs*17), with a history of breast cancer in her late 40 s, ovarian cancer in her late 50 s and ovarian recurrence in her mid-60 s. As part of her cancer treatment, the participant had previously undergone a total abdominal hysterectomy with bilateral salpingo-oophorectomy, and as part of her clinical care, was receiving monthly CA125 screening, quarterly Chest-Abdomen-Pelvis (CAP)-CT, and annual breast MRI and mammograms. Results of all the above clinical screening investigations for >3 years from the time of study enrollment showed no evidence of malignancy. The participant was on olaparib at the time of enrollment.

#### CHARM2 liquid biopsy findings

sWGS identified genome-wide copy number changes, with a tumor fraction of 11.5%. The result was disclosed to the participant 51 days after the blood sample was provided. The participant provided a follow-up confirmatory CHARM2 blood sample (completed 2 months after the bloodwork for the initial positive result) that was also positive (tumor fraction of 13.6%).

#### Follow-up workflow

**Fig. 2:**
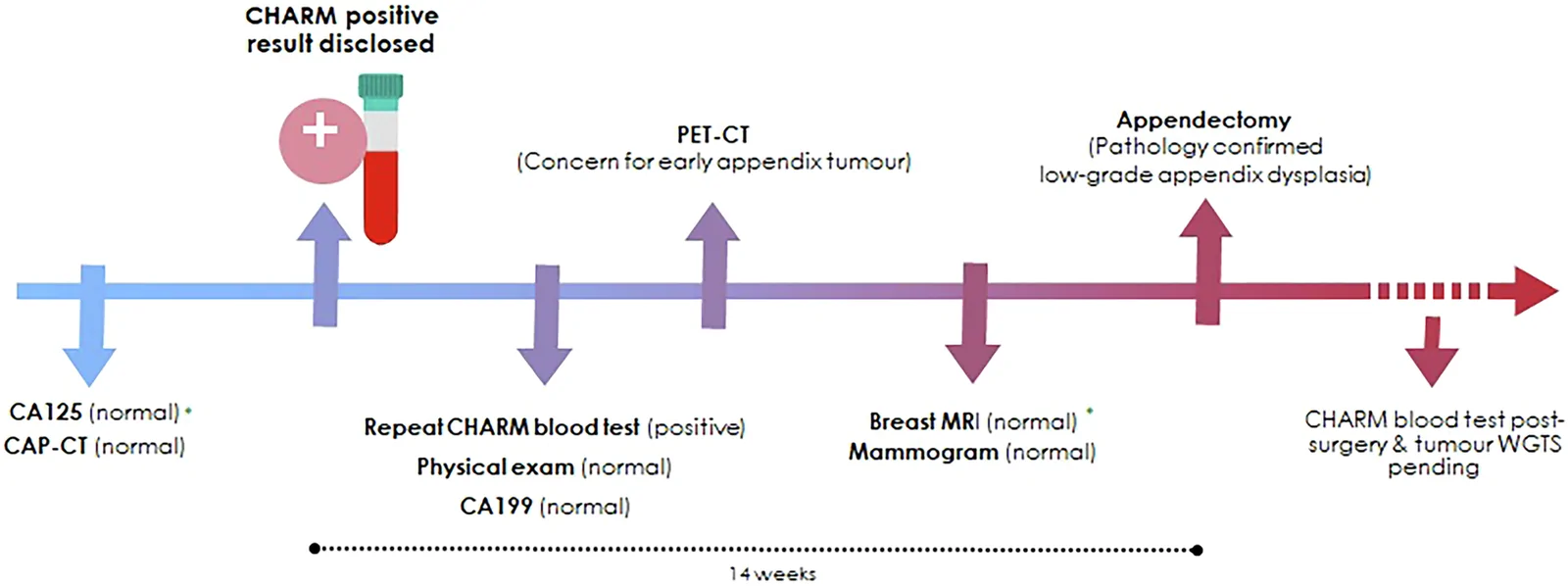
Summary of the follow-up investigations completed for CHARM2 positive case 1. * indicates the investigations completed as part of the participant’s standard of care. CAP-CT: Chest-Abdomen-Pelvis CT.

The participant attended the genetics clinic at UHN for a physical exam, with normal findings. In accordance with the follow-up protocols, due to her history of breast cancer, the participant underwent a PET-CT scan (completed 3 weeks after the result was returned) to rule out metastasis. The PET-CT identified a bilobed metabolically active mass in the appendix, which was concerning for an early appendiceal tumor. The participant underwent an appendectomy, and subsequent pathology identified a low-grade appendix dysplasia (Fig. 2). A blood sample was collected 1.5 months after appendectomy to assess copy number changes using cfDNA sequencing. Evidence of cancer-derived fragments in the copy number track still remained, although the purity of 7.7% for this sample was below the ctDNA positive threshold of 10% for CNV. Furthermore, the participant was referred to hematology for follow-up on concerns for clonal hematopoiesis of indeterminate potential (CHIP). Current workup shows macrocytic anemia, likely secondary to reticulocytosis post-splenectomy, as well as their olaparib treatment.

#### Next steps

Whole-genome tumor sequencing (WGTS) of the appendix tumor is underway to further characterize the cfDNA signal.

### Positive case 2

#### Demographics and clinical background

A female in her mid-50 s with LS and a germline *PMS2* variant in c.736_741delinsTGTGTGTGAAG; p.Pro246Cysfs*3 with a history of breast cancer in her early 50 s (invasive lobular carcinoma, T2 N1a, ER/PR + , HER2-), and multiple sclerosis. As part of her cancer treatment, the participant had previously undergone a bilateral mastectomy. Due to her LS diagnosis, the participant has also undergone a total abdominal hysterectomy and bilateral salpingo-oophorectomy. As part of her clinical screening, the participant was undergoing annual colonoscopies; however, at the time of the CHARM2 result disclosure, the participant was overdue for a colonoscopy, having had her last surveillance 20 months before.

#### CHARM2 liquid biopsy findings

The targeted panel identified a somatic mutation in a microsatellite region within the *NF1* gene (c.2160del; p.Cys721Valfs*27), at a variant allele fraction of 3%. The somatic mutation is located in an MSI site, in keeping with the participant’s LS diagnosis. The result was disclosed to the participant 62 days after the blood sample was provided. The participant provided a follow-up confirmatory CHARM2 blood sample (completed two months after the bloodwork for the initial positive result) that was also positive (*NF1* somatic mutation VAF at 1%). Interestingly, a third CHARM2 blood sample collected four months after the initial positive result identified a somatic *CDH1* mutation (c.1843A>C; p.Ile615Leufs*16), in addition to the previously identified *NF1* somatic mutation, also located in an MSI site (Supplementary Fig. S19).

#### Follow-up workflow

**Fig. 3:**
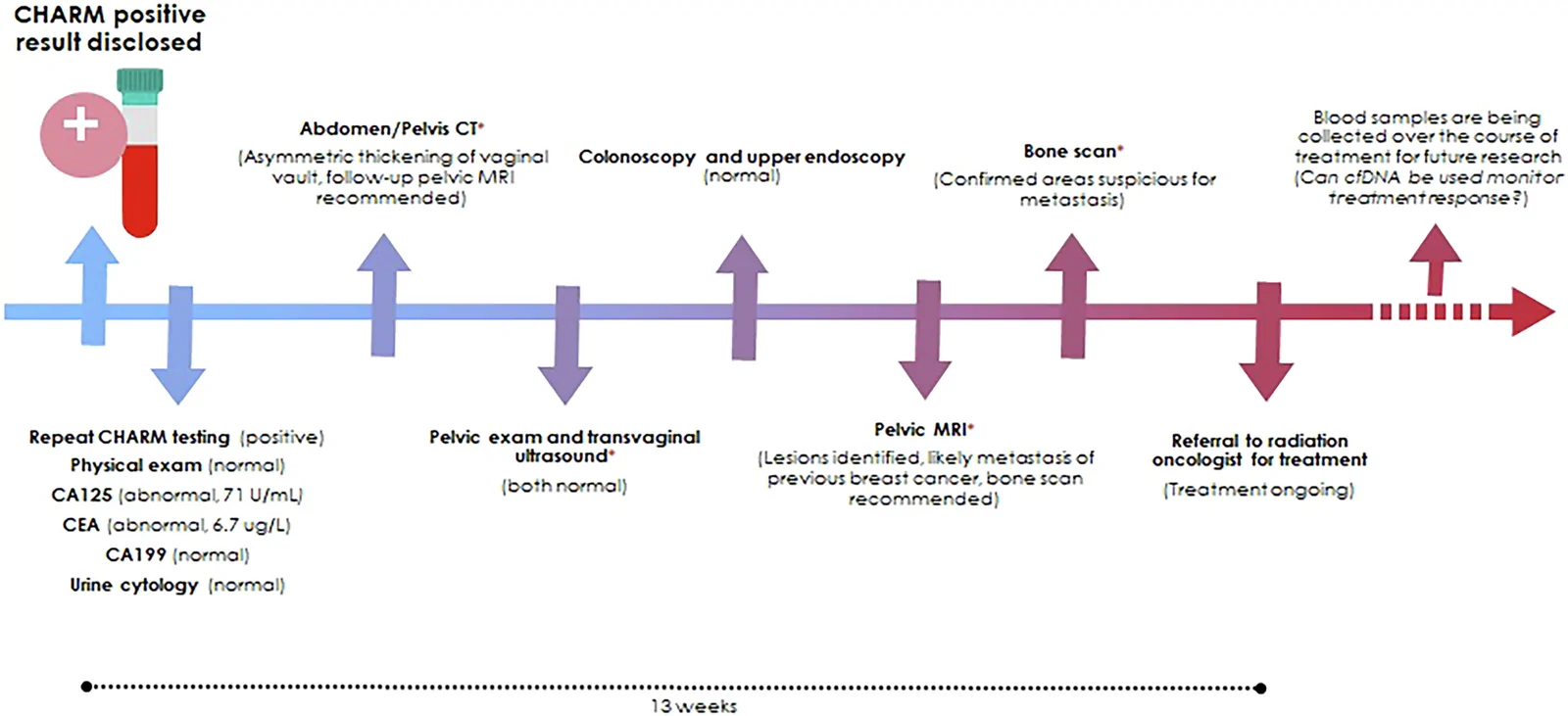
Summary of the follow-up investigations completed for CHARM2 positive case 2. * indicates the investigations completed in addition to those in the follow-up workflow, due to elevated CA125 level.

The participant attended the genetics clinic at UHN for a physical exam, with normal findings. In accordance with the follow-up protocols, at the time of her in-person appointment, tumor markers (CA199, CA125, and CEA) and urine cytology were completed, and a colonoscopy and upper endoscopy were ordered. Due to elevated CA125 levels, an Abdomen-Pelvis CT was immediately ordered, which identified asymmetric thickening of the vaginal vault. Follow-up investigations for the CT finding included a pelvic exam, transvaginal ultrasound, and pelvic MRI. Pelvic MRI identified bone metastasis in the proximal left femur, concordant on bone scan, consistent with recurrent breast cancer. Brain MRI did not identify any sites of metastasis. It is unlikely the symptomless bone metastasis would have come to attention at this time without cfDNA analysis, as it is rare for ongoing bone metastasis surveillance to be conducted. The patient completed one round of radiation therapy to her left femur and is currently undergoing systemic therapy (ribociclib and letrozole) (Fig. 3).

#### Next steps

The CHARM2 study is collecting blood samples over the course of the participant’s treatment (every two to three months). These samples are being banked with the CHARM Biobank for future research.

### Positive case 3

#### Demographics and clinical background

A female in her mid-40 s with a germline *BRCA1* variant, c.1350del; p.Lys450Asnfs*3, with a history of fallopian tube cancer in her early 40 s and recurrence shortly after. As part of her cancer treatment, the participant had previously undergone a total abdominal hysterectomy and bilateral salpingo-oophorectomy. Due to her *BRCA1* status, she also had a bilateral mastectomy. As part of her previous cancer monitoring, the participant was having annual CAP-CTs and annual CA125 tests, and no new symptoms suggestive of recurrence were reported. The participant was on olaparib at the time of enrollment.

#### CHARM2 liquid biopsy findings

sWGS identified genome-wide copy number changes, with a tumor fraction of 11.5%. The result was disclosed to the participant 50 days after the blood sample was provided. The participant provided a follow-up confirmatory CHARM2 blood sample (completed two months after the bloodwork for the initial positive result) that showed a tumor fraction of 7.5%, which is below the cfDNA positive threshold of 10% for CNVs. However, comparison of the copy number results found a near-identical pattern of gains and losses between the two timepoints, and therefore, a positive clinical report was issued for the repeat testing.

#### Follow-up workflow

**Fig. 4:**
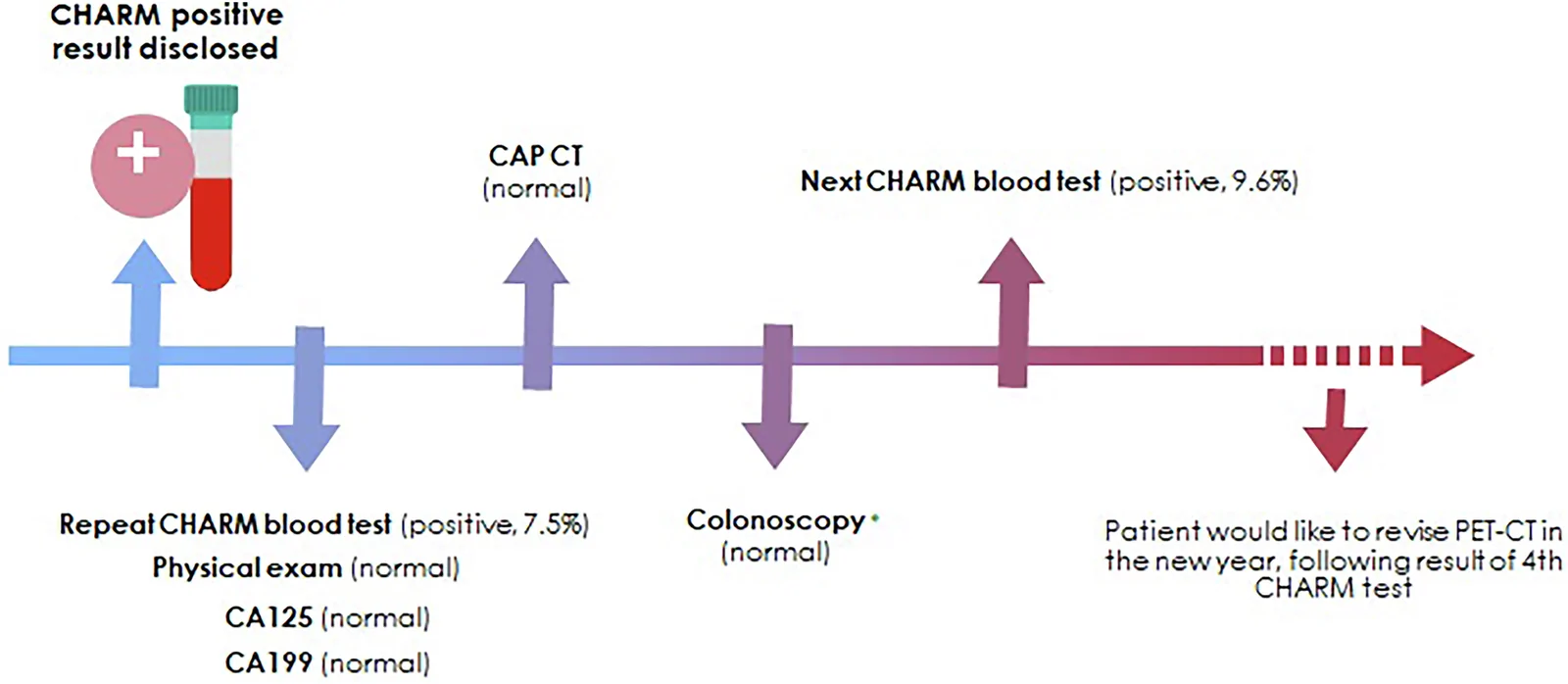
Summary of the follow-up investigations completed for CHARM2 positive case 3. * indicates the investigations completed as part of the participant’s clinical care. CAP-CT: Chest-Abdomen-Pelvis CT.

The participant attended the genetics clinic at UHN for a physical exam, with normal findings. In accordance with the follow-up protocols, at the time of her in-person appointment, tumor markers were completed (CA125, CA199), and a CAP-CT was ordered. Though the participant was getting CAP-CTs as part of her clinical follow-up, at the time of the positive CHARM2 study result, the participant’s last CAP-CT was 10 months prior, and therefore, one was ordered by the research team. The CAP-CT did not show any evidence of cancer. The participant also completed a colonoscopy as part of her routine cancer screening, which showed no abnormalities (Fig. 4). In accordance with the protocol, the participant has provided her third CHARM2 blood sample for cfDNA testing, which was also positive and showed a tumor fraction of 9.6%.

#### Next steps

The participant was informed of the third positive result. The participant expressed continued interest in the CHARM2 study with serial cfDNA sampling and analysis, but declined PET-CT.

### False negative case 1

A *BRCA2* positive patient in her early 60 s, with a germline variant of c.4965 C > G; p.Tyr1655*, a history of a total abdominal hysterectomy and bilateral salpingo-oophorectomy, and no cancer history. The participant provided her first CHARM2 blood sample one month prior to her routine breast screening (mammogram and breast MRI). Her routine mammogram showed no abnormalities; however, her breast MRI identified a 7 mm area of enhancement. Subsequent biopsy confirmed stage 1 invasive ductal carcinoma, a 1.6 mm tumor size, ER 10% + /PR-/HER2-. Her CHARM2 cfDNA testing did not identify a cancer signal. The participant declined to provide additional blood samples.

### False negative case 2

A *BRCA2* positive patient in her early 70 s, with a germline variant of c.574_575delAT; p.Met192Valfs*13, and a history of left breast cancer about 20 years ago, treated with mastectomy, chemotherapy, and radiation therapy. The participant had provided three CHARM2 blood samples, all of which resulted in cfDNA being negative. Three months after their third negative result, the participant was experiencing abdominal discomfort and underwent a standard-of-care CAP-CT. This identified a pancreatic mass and multiple peritoneal nodules, which were biopsied and confirmed to be metastatic carcinoma, likely of pancreatic origin. Tumor sequencing request of the biopsy tissue is currently underway. The participant declined to provide additional blood samples.

## Future directions and clinical implementation

The CHARM2 trial is predicted to be seven years in length (2024-2031), encompassing two years of recruitment, four years of blood collection per participant, and one year of follow-up to collect medical history. A main challenge we have encountered is sample failure due to lower blood plasma levels, resulting in insufficient cfDNA yields. These participants are asked to go for a repeat collection, which normally yields sufficient cfDNA levels to proceed with the testing.

The strength of our randomized controlled trial in assessing cfDNA for cancer early detection is the enrollment of individuals with germline gene variants, enabling the evaluation of liquid biopsy in a practical way. Beginning in 2026 and 2027, we plan to interview 50–60 participants in the intervention arm to further explore their experiences with standard of care screening, perceived value of cfDNA testing and ways to optimize implementation in practice. We will also interview 10 providers who interfaced with the study to explore experiences and ways to optimize clinical implementation. We will use purposeful maximal variation sampling [68] to capture as broad a range of views and experiences as possible across age, sex, discipline, length of time in practice, institutional setting, and geographic location.

To guide clinical implementation, we plan to develop a survey to measure the value of ctDNA information in HCS management decisions using a discrete choice experiment study design. This survey will be distributed to participants in this trial and will be developed based on the qualitative interviews completed as part of the trial. Additionally, we will conduct an economic analysis to quantify the potential economic impact of early detection of cancer in high-risk patients through routine profiling of cfDNA compared to the current standard of care using data from this trial and published literature, reported as an incremental cost-effectiveness ratio. It is also anticipated that sequencing costs will decrease over time.

Other factors supporting clinical implementation of the cfDNA test include our use of community laboratories to ensure equitable access. Clinical implementation of a blood test, particularly using community laboratories, will improve access as screening is completed at major hospitals and impacted by location and availability of imaging machines. Furthermore, in our questionnaire, we ask participants to reflect on their satisfaction with the study's blood test frequency and will examine patient preferences for test frequency, as well as participant compliance with blood collections at least three times a year over the duration of the trial (i.e., testing fatigue), prior to clinical implementation. Some participants have expressed that although they were very interested in the trial, they did not enroll due to the high frequency of blood collections. Finally, one of the aims of our trial is to learn from the fragmentomics, methylation, and additional data to guide testing and incorporate methylation into future iterations of the cfDNA test.

Previous implementation of an MCED test in a tertiary ambulatory internal medicine clinic required extensive physician and patient education, integration with the institutional electronic health record and standardized order, and a clinical leadership task force to interpret cancer signal detected results [69]. In the NHS-Galleri trial, the existing e-referral service was used in combination with a new e-referral form for nurses to refer trial participants directly into secondary care and to standardize information, minimizing clinical care burden [70]. We intend to follow these same principles and guidelines to clinically implement our liquid biopsy early detection test in a healthcare setting.

## Conclusions

The detection and monitoring of active cancer through MCED tests is a rapidly evolving application of cfDNA sequencing technologies. Our focus on conducting a randomized controlled trial on individuals with HBOC, LS, LFS, NF1 and CDH1 illustrates the utility of cfDNA in inherited cancer, while assessing the clinical effectiveness of cfDNA testing as a mechanism for cancer early detection. This remains a unique approach due to our ability to identify and serially bank blood samples from HCS populations over multiple years, made possible by our deep and ongoing engagement with cancer clinics across Canada and the use of community blood laboratories for equitable access. This trial is a crucial next step towards better management for individuals at risk of developing cancer and the clinical implementation of liquid biopsy early detection tests in patient care.

## Supplementary information

Supplementary information

## Data Availability

CHARM has established the Phenomic Liquid Biopsy Resource (PLBR) to share genome-wide fragment data, allowing for fragmentomic and methylation analysis to improve collaboration (https://fragmentomics.ca). We developed the oncographer database for standardized clinical data abstraction using the Marathon of Hope Cancer Centers Network (MOHCCN) standard (https://mcoder2.ca/). CHARM is committed to abstracting clinical data to MOHCCN clinical data standards to ensure consistency of cancer data available to clinicians and researchers. MOHCCN standards include elements from the International Cancer Genome Consortium Accelerating Research in Genomic Oncology (ICGC-ARGO), other unique MOHCCN elements, and the American Society of Clinical Oncology Minimal Common Oncology Data Elements (ASCO mCODE) data dictionaries, which define each data element that is collected. The genetic data that support the findings of this study have been submitted to be made publicly available in ClinVar (https://www.ncbi.nlm.nih.gov/clinvar/). Accession numbers are SCV007127685, SCV007127686, SCV007127687, SCV007127688, SCV007127689, SCV007127690, and SCV007127691.
