# Supplementary material for: Non-invasive screening in hereditary cancer: a randomized controlled trial to test cell-free DNA-based early detection in the CHARM consortium
Source: Eur J Hum Genet. 2026 Jan 29;34(9):1243–53. doi: 10.1038/s41431-026-02014-z (PMC13550592; doi:10.1038/s41431-026-02014-z)
Supplement: Supplementary file 1 [file 41431_2026_2014_MOESM1_ESM.pdf]

Summary: Supplementary figures (S1-S19) included as a single combined PDF document. Includes life cycle of an individual with a hereditary cancer syndrome, CHARM consortium map, workflows of the clinical follow-up for individuals with a positive cell-free DNA result, and mutations in the microsatellite region for one of the participants.

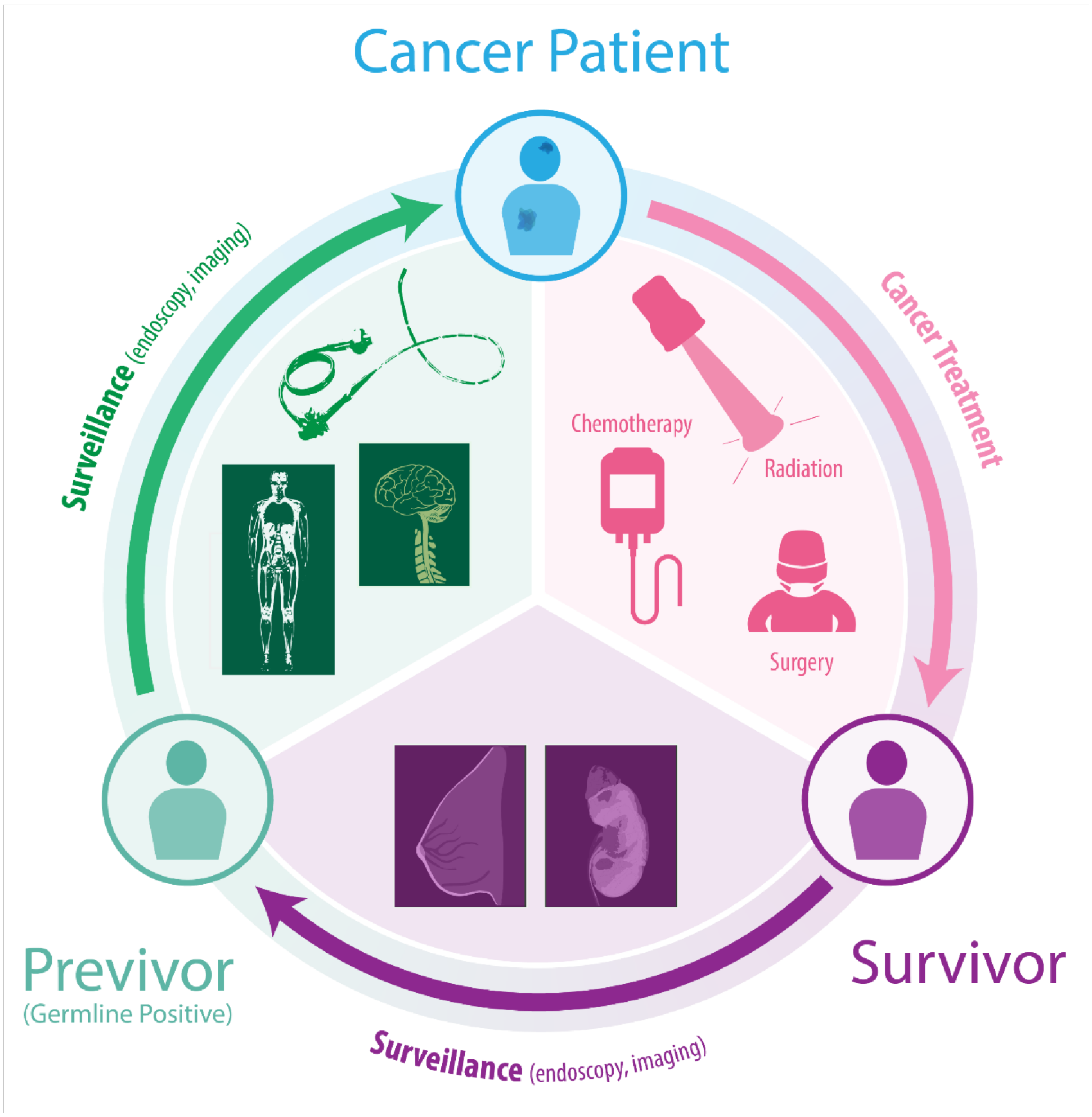

Supplemental Figure S1. Lifecycle of an individual with hereditary cancer.

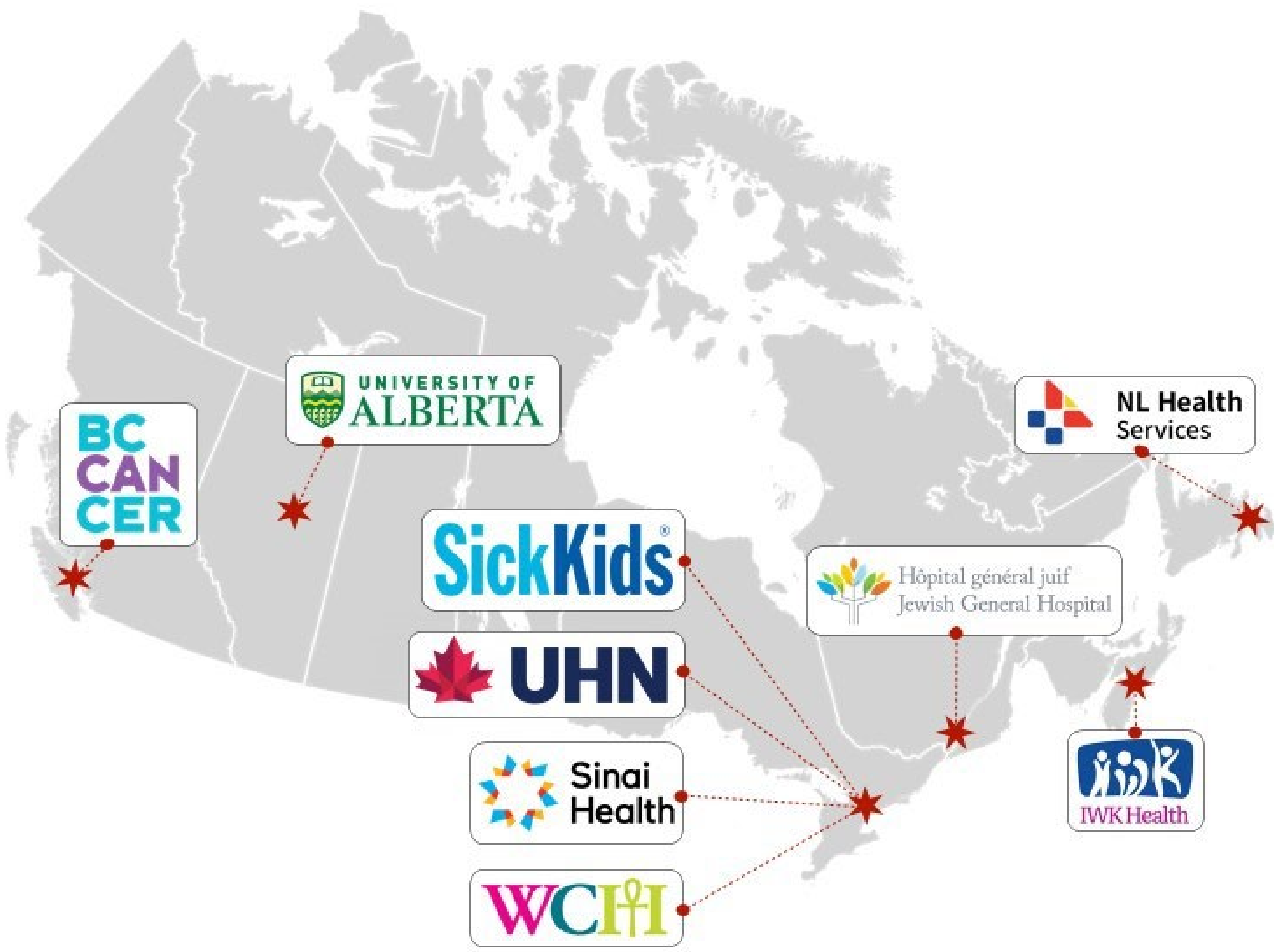

Supplemental Figure S2. Map of Canada depicting sites recruiting participants in the CHARM consortium.

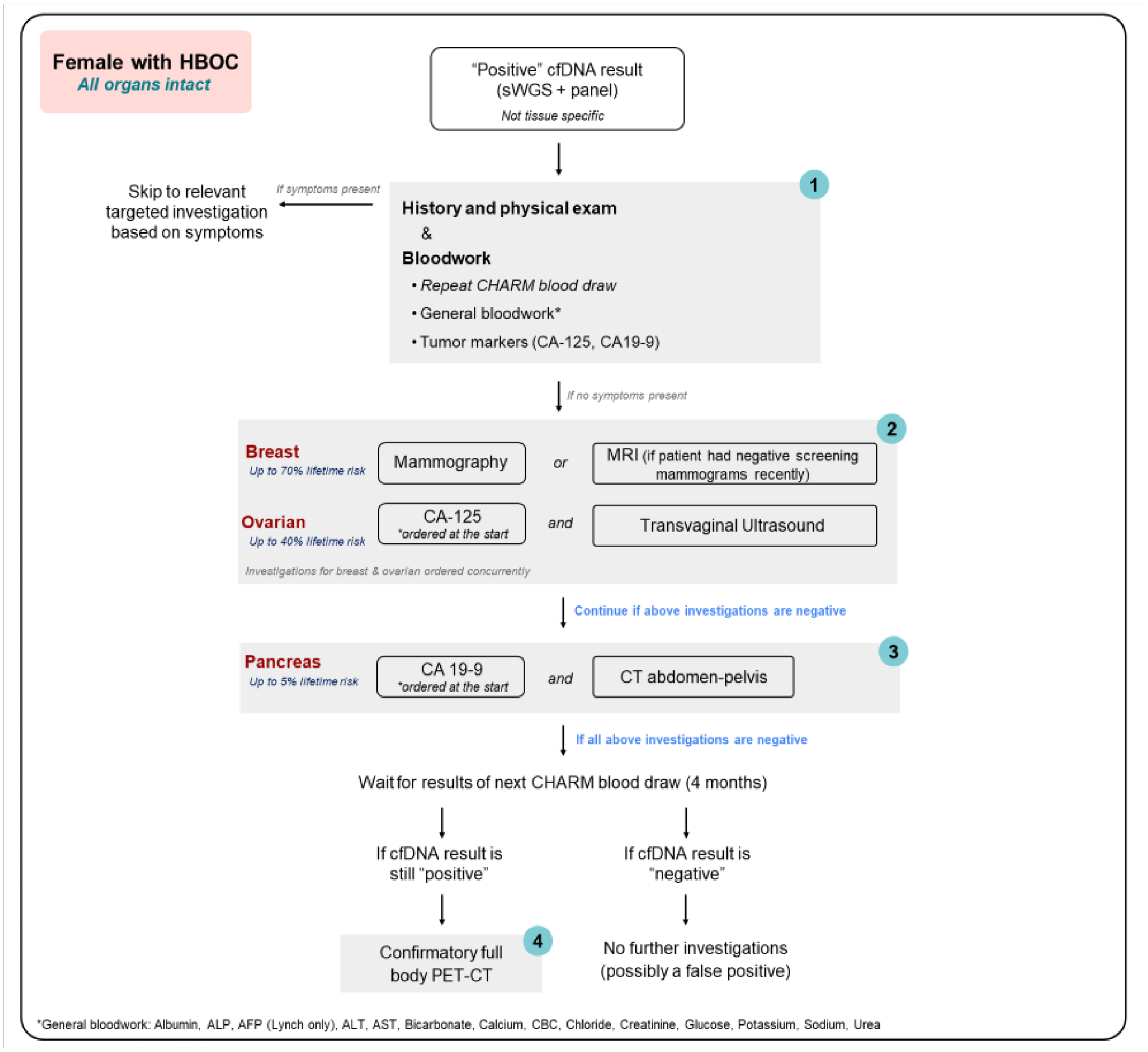

Supplemental Figure S3. Clinical follow-up workflow for Hereditary Breast and Ovarian Cancer (HBOC) cohort *BRCA1/2* heterozygote with all organs intact.

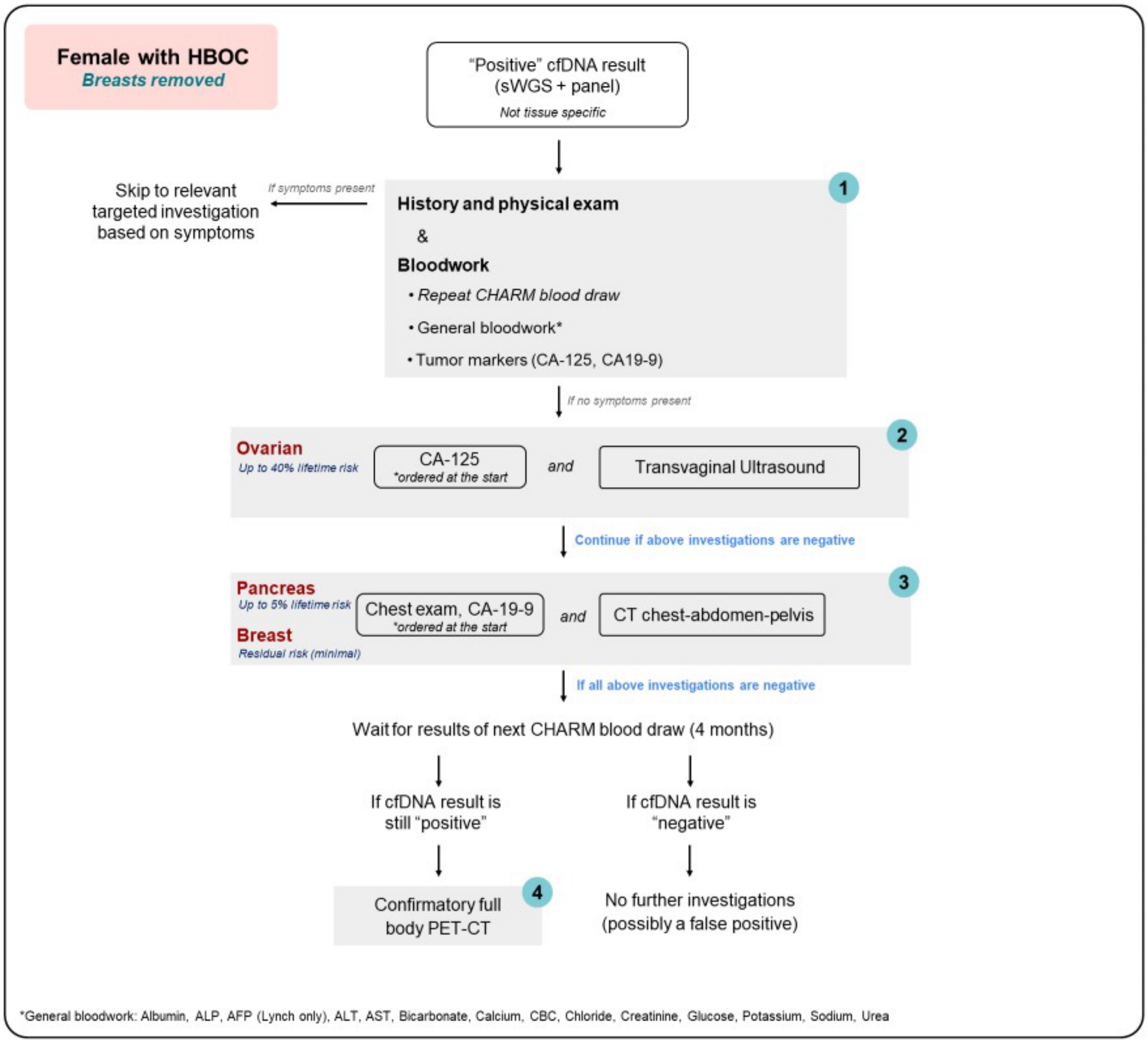

Supplemental Figure S4. Clinical follow-up workflow for Hereditary Breast and Ovarian Cancer (HBOC) cohort *BRCA1/2* heterozygote for a female participant with breasts removed.

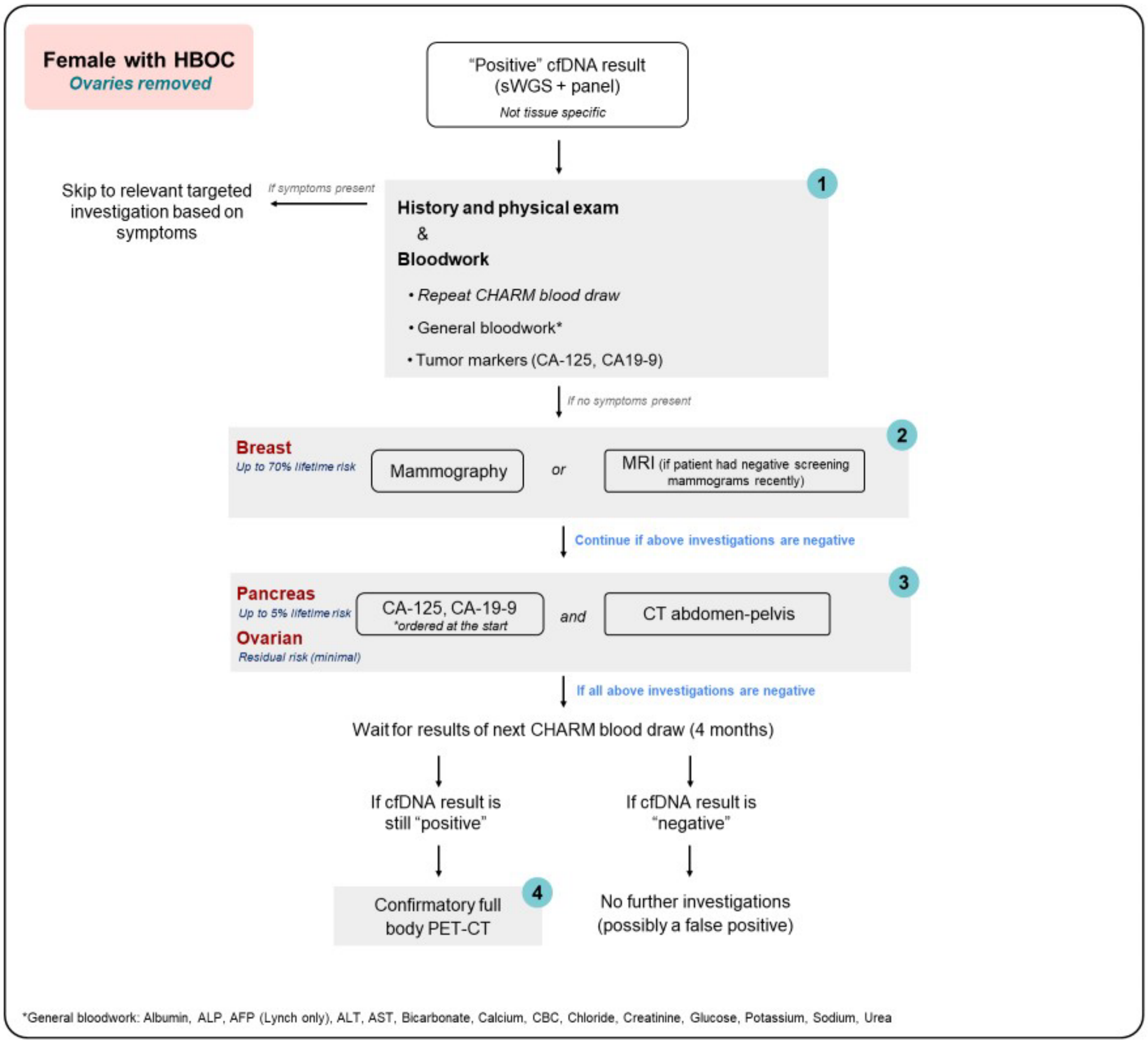

Supplemental Figure S5. Clinical follow-up workflow for Hereditary Breast and Ovarian Cancer (HBOC) cohort *BRCA1/2* heterozygote for a female participant with ovaries removed.

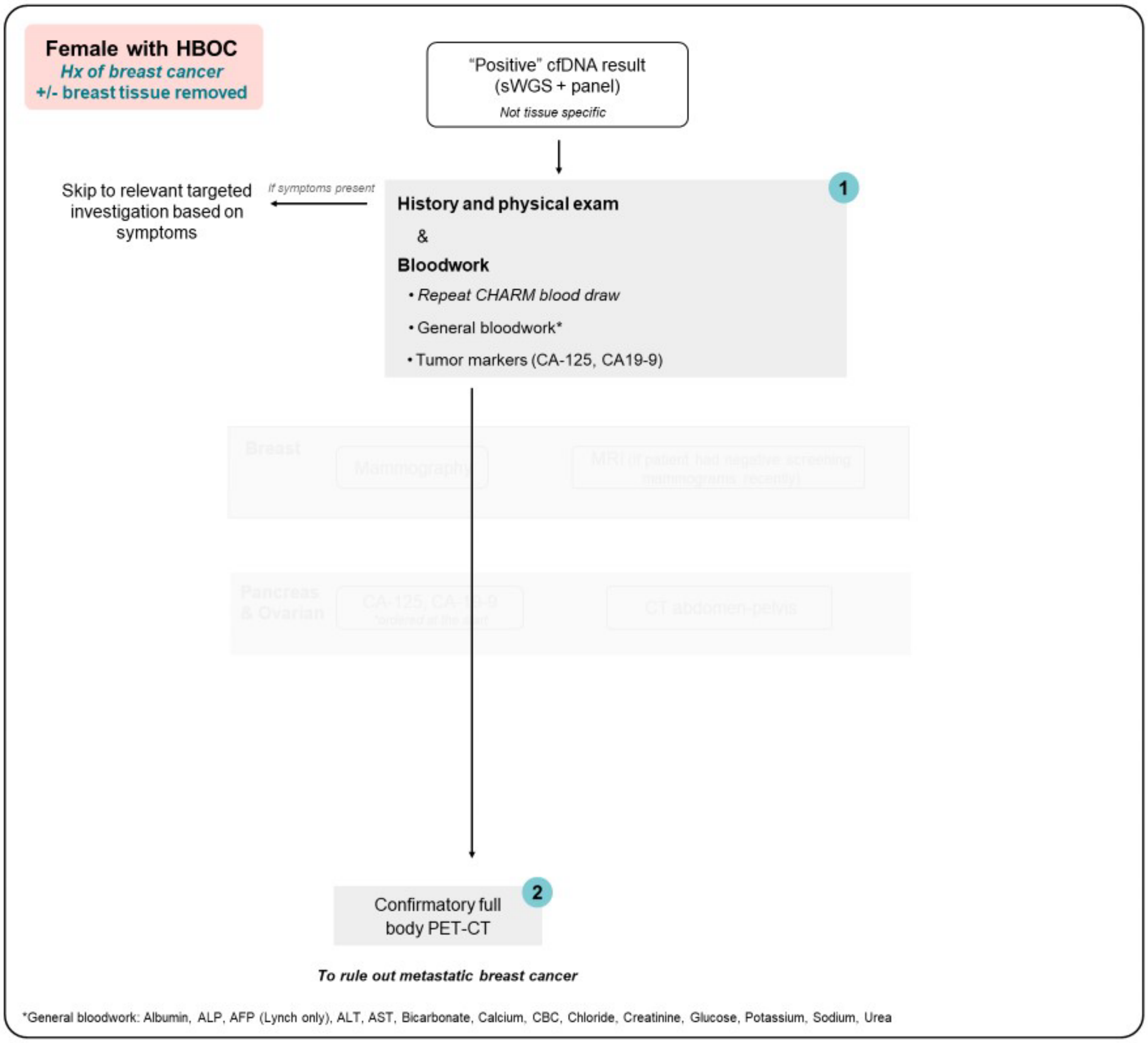

Supplemental Figure S6. Clinical follow-up workflow for Hereditary Breast and Ovarian Cancer (HBOC) cohort *BRCA1/2* heterozygote for a female participant with a history of breast cancer (with or without breasts removed).

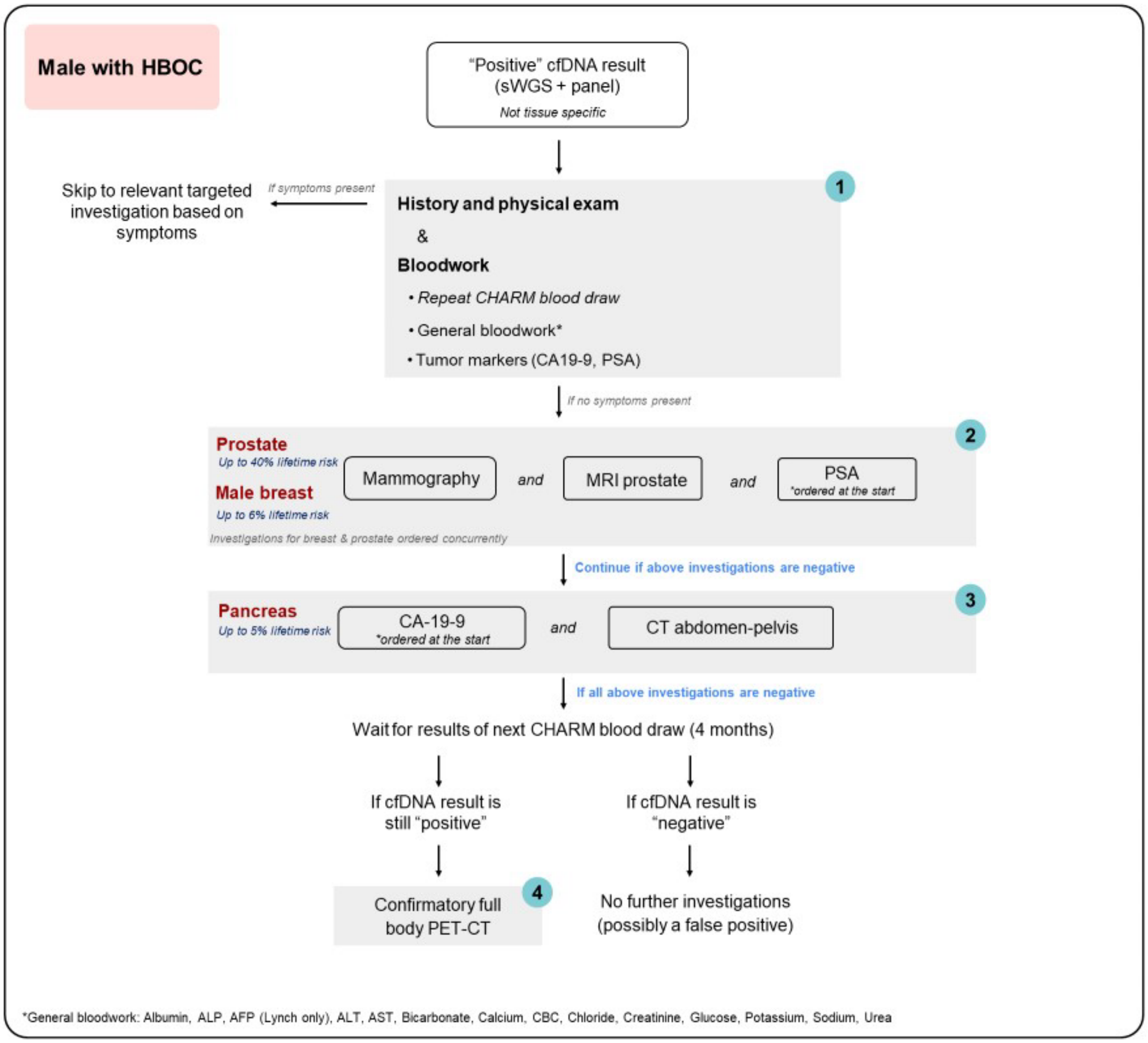

Supplemental Figure S7. Clinical follow-up workflow for Hereditary Breast and Ovarian Cancer (HBOC) cohort *BRCA1/2* heterozygote for a male participant.

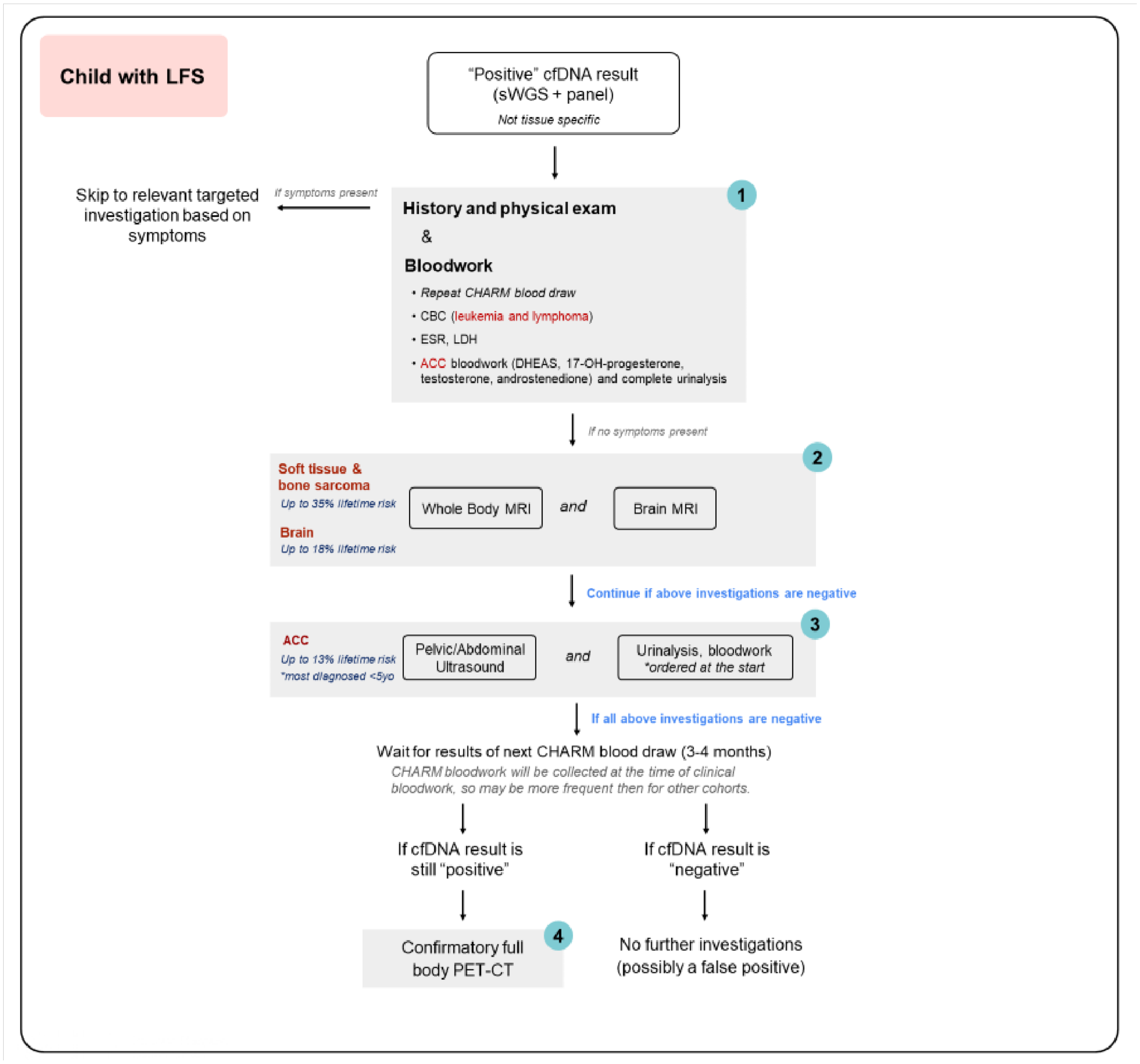

Supplemental Figure S8. Clinical follow-up workflow for Li-Fraumeni Syndrome (LFS) cohort for a child with LFS.

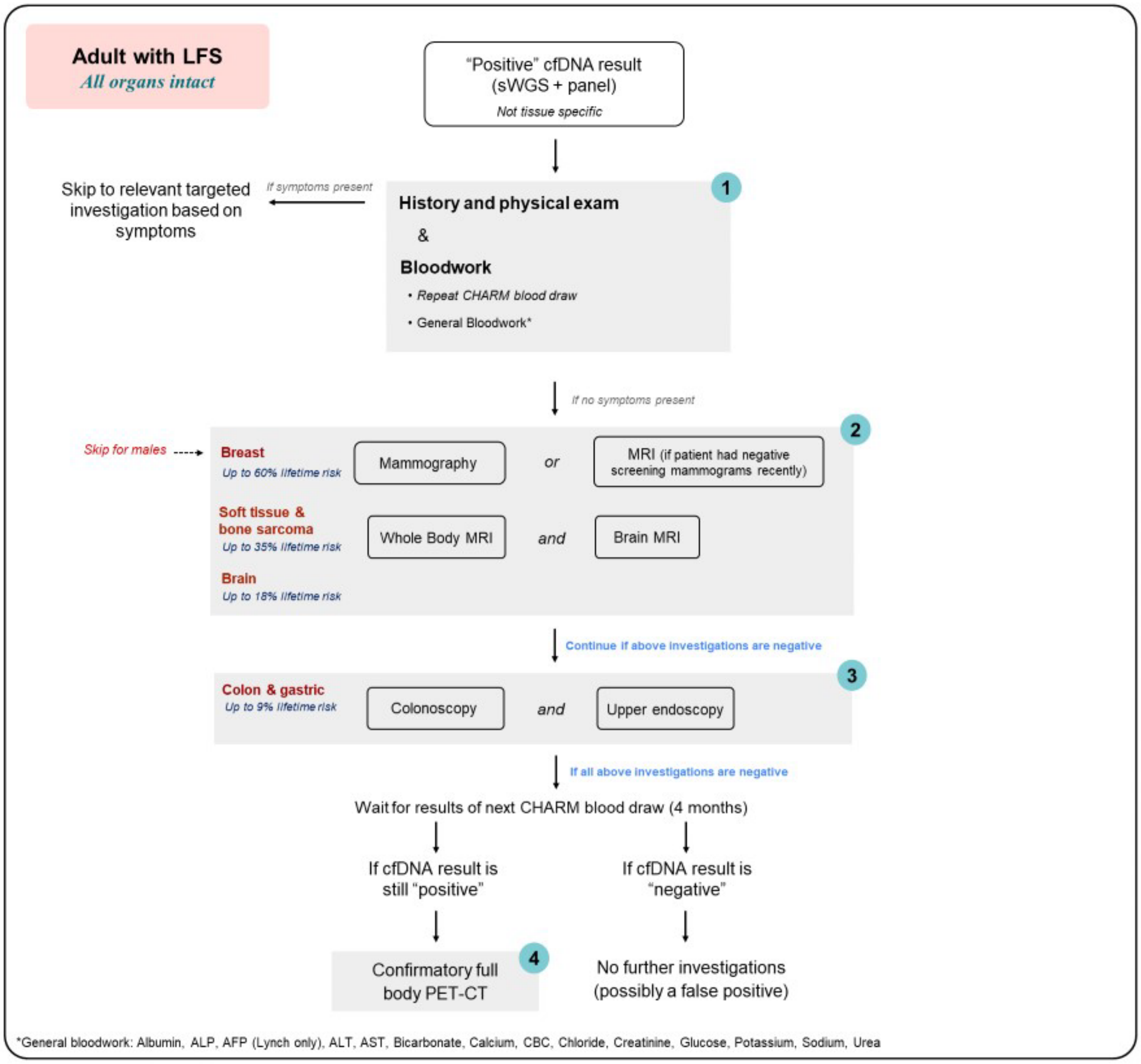

Supplemental Figure S9. Clinical follow-up workflow for Li-Fraumeni Syndrome (LFS) cohort for an adult with LFS and all organs intact.

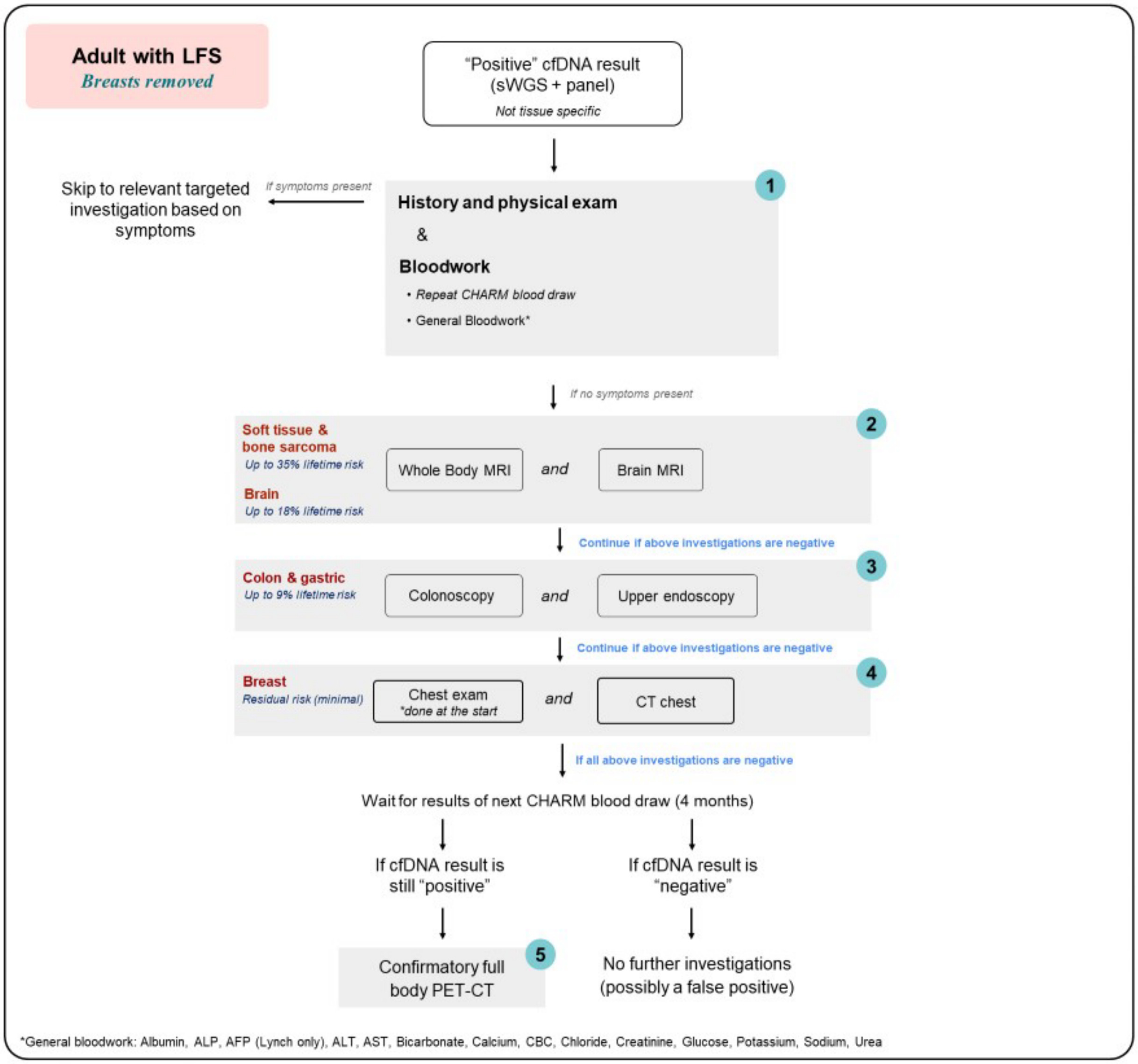

Supplemental Figure S10. Clinical follow-up workflow for Li-Fraumeni Syndrome (LFS) cohort for an adult with LFS and breasts removed.

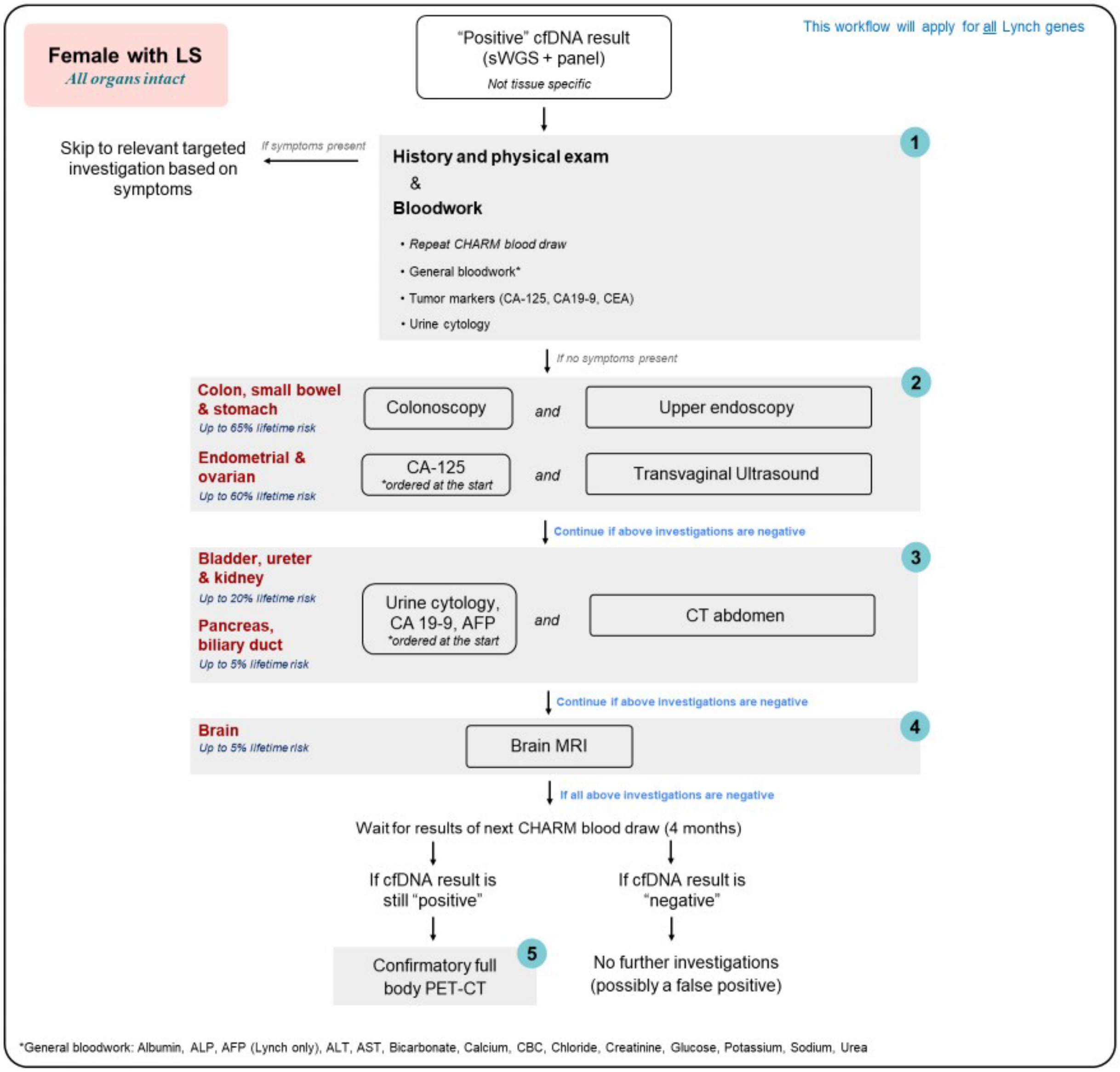

Supplemental Figure S11. Clinical follow-up workflow for Lynch Syndrome (LS) cohort for a female with LS, any pathogenic/likely pathogenic variant, and all organs intact.

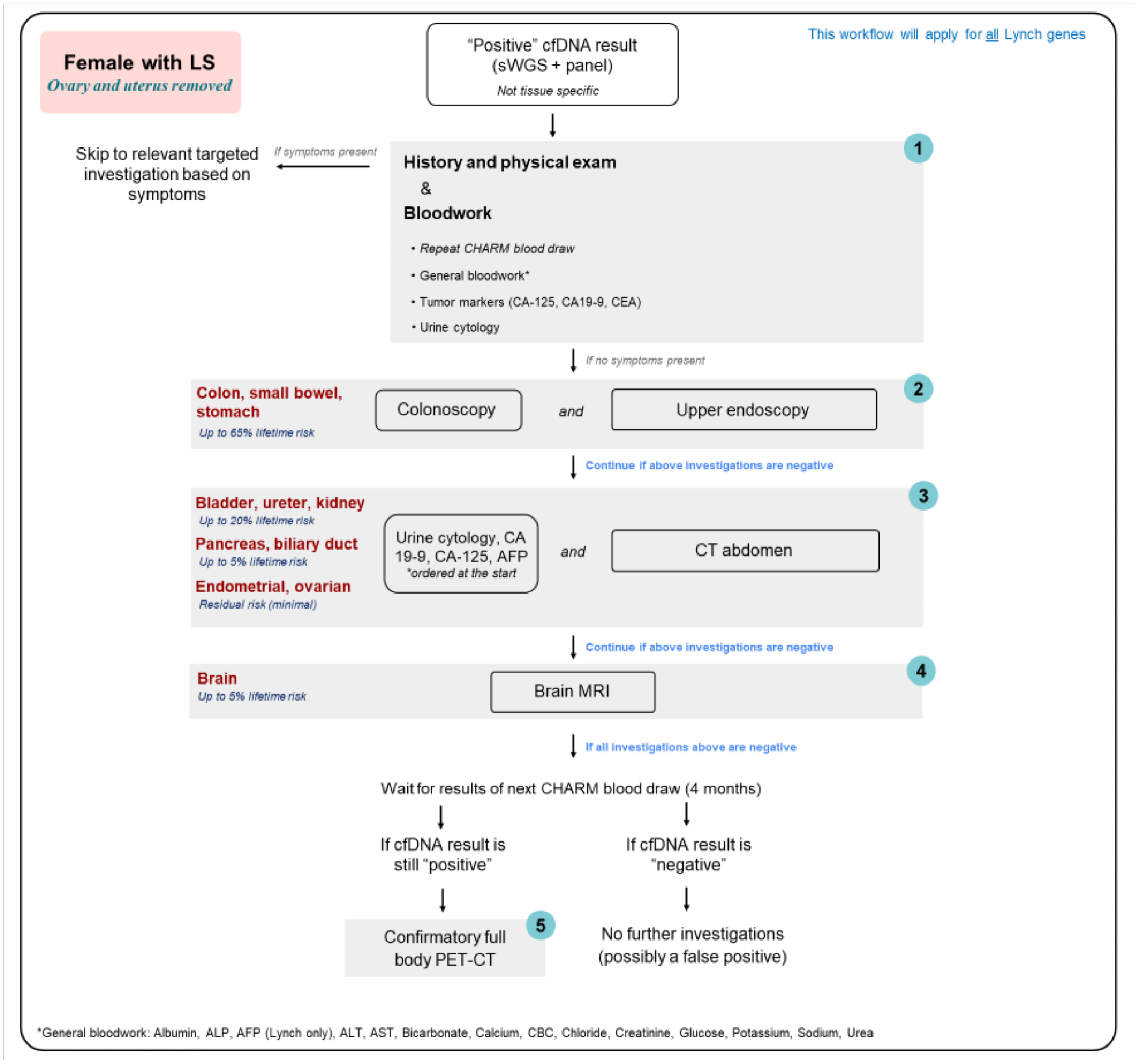

Supplemental Figure S12. Clinical follow-up workflow for Lynch Syndrome (LS) cohort for a female with LS, any pathogenic/likely pathogenic variant, with ovary and uterus removed.

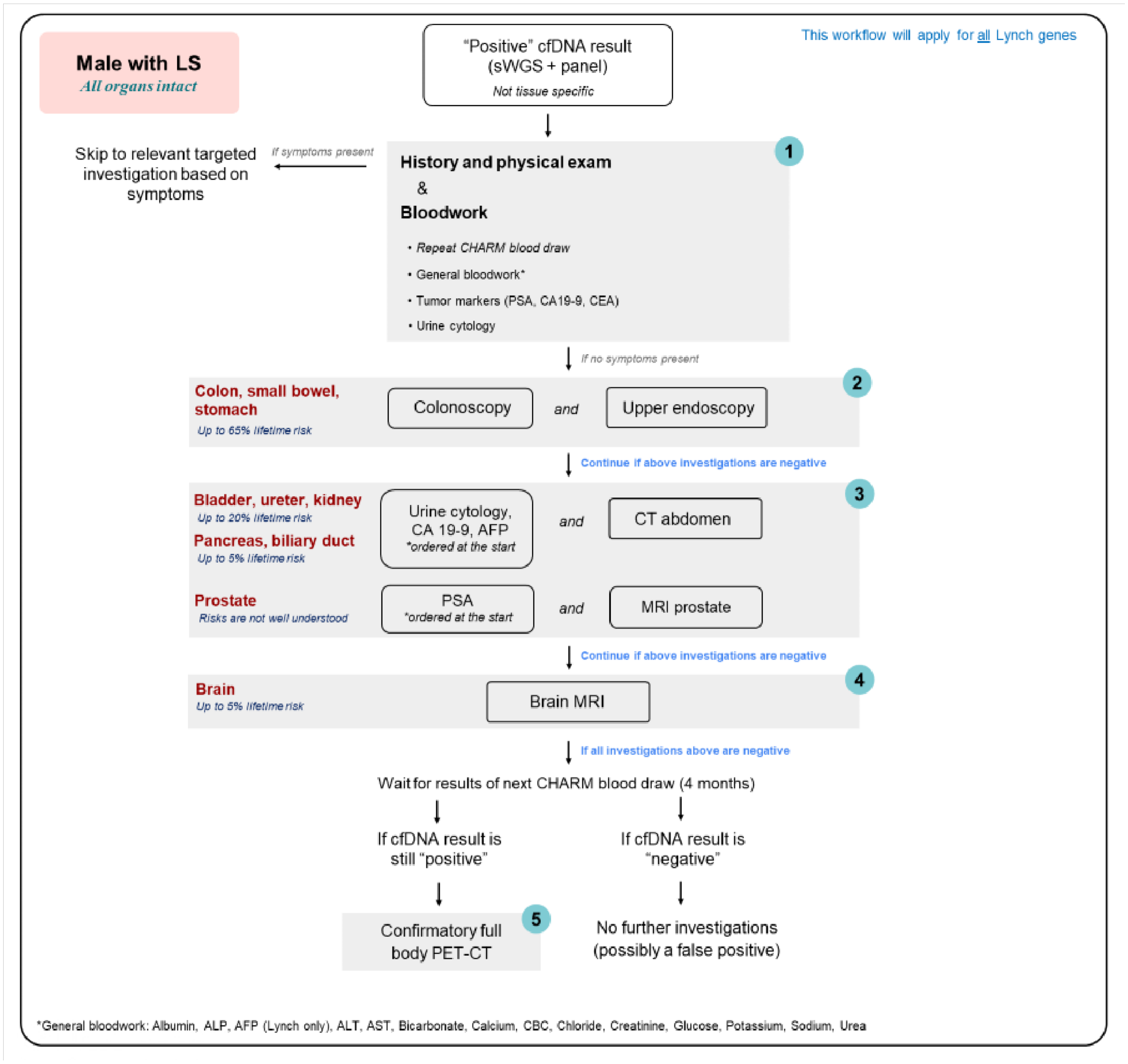

Supplemental Figure S13. Clinical follow-up workflow for Lynch Syndrome (LS) cohort for a male with LS, any pathogenic/likely pathogenic variant, and all organs intact.

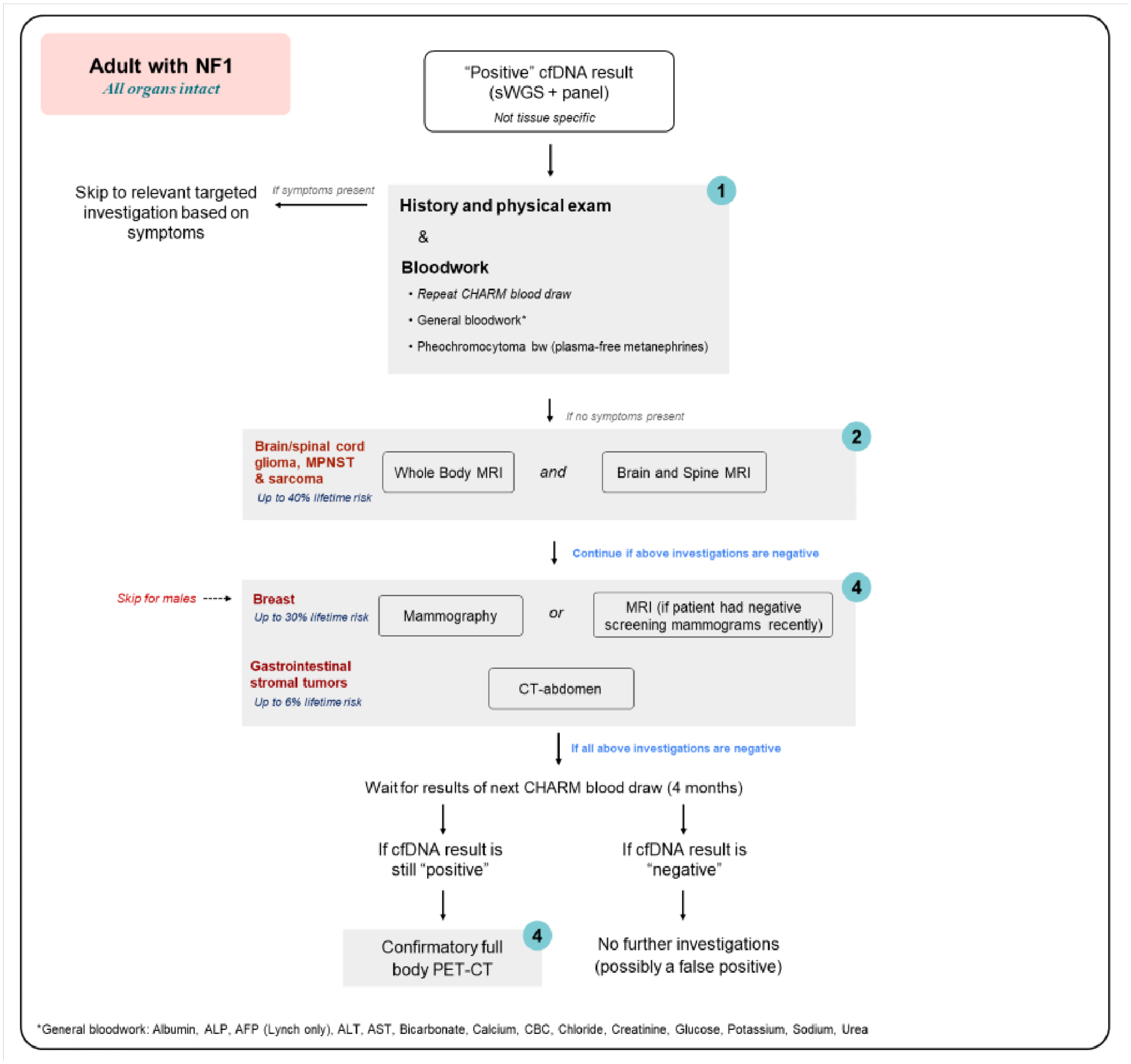

Supplemental Figure S14. Clinical follow-up workflow for Neurofibromatosis Type 1 (NF1) cohort for all adults with NF1 and all organs intact.

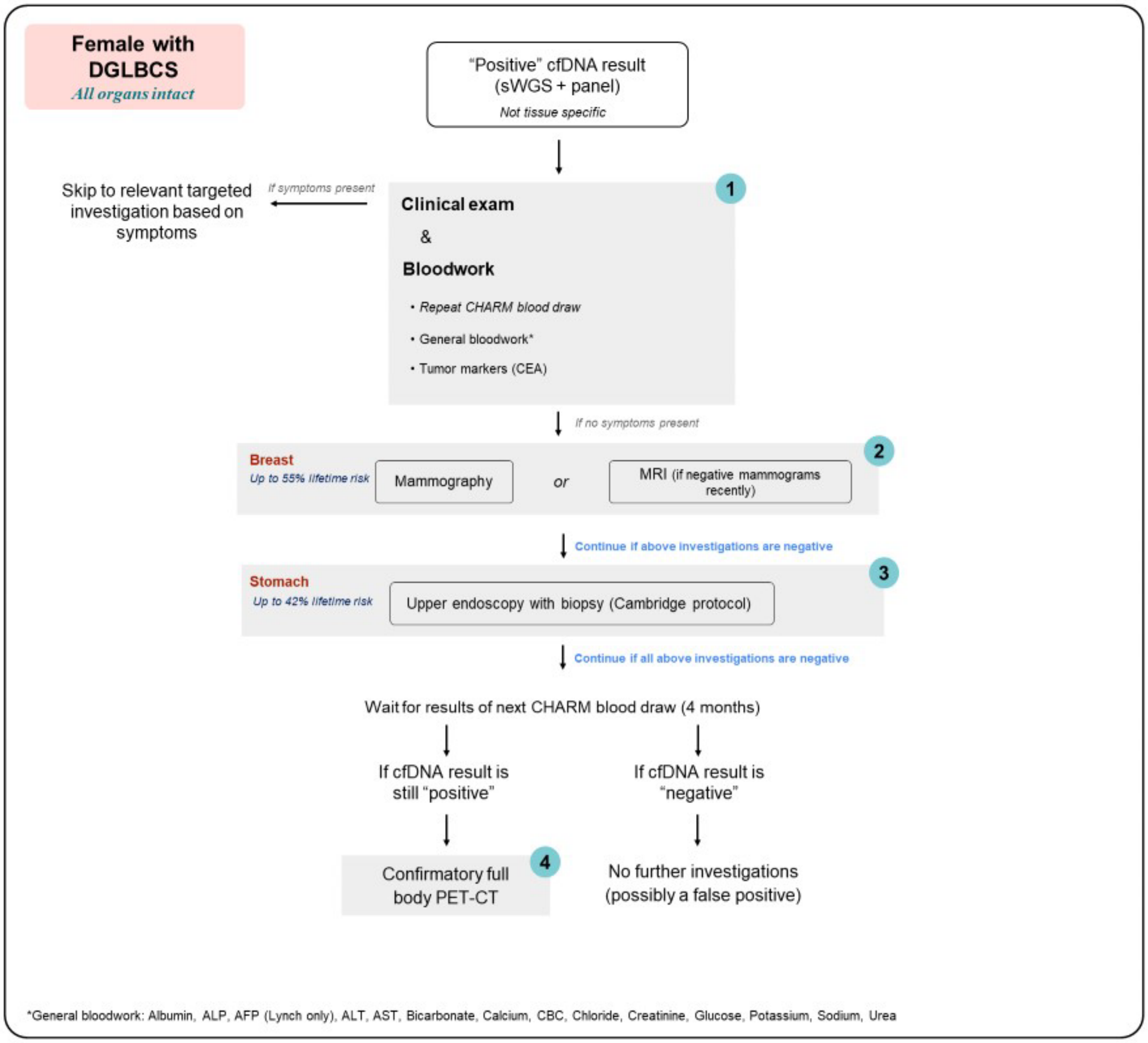

Supplemental Figure S15. Clinical follow-up workflow for Diffuse Gastric and Lobular Breast Cancer syndrome (DGLBCS) cohort for female participants with all organs intact.

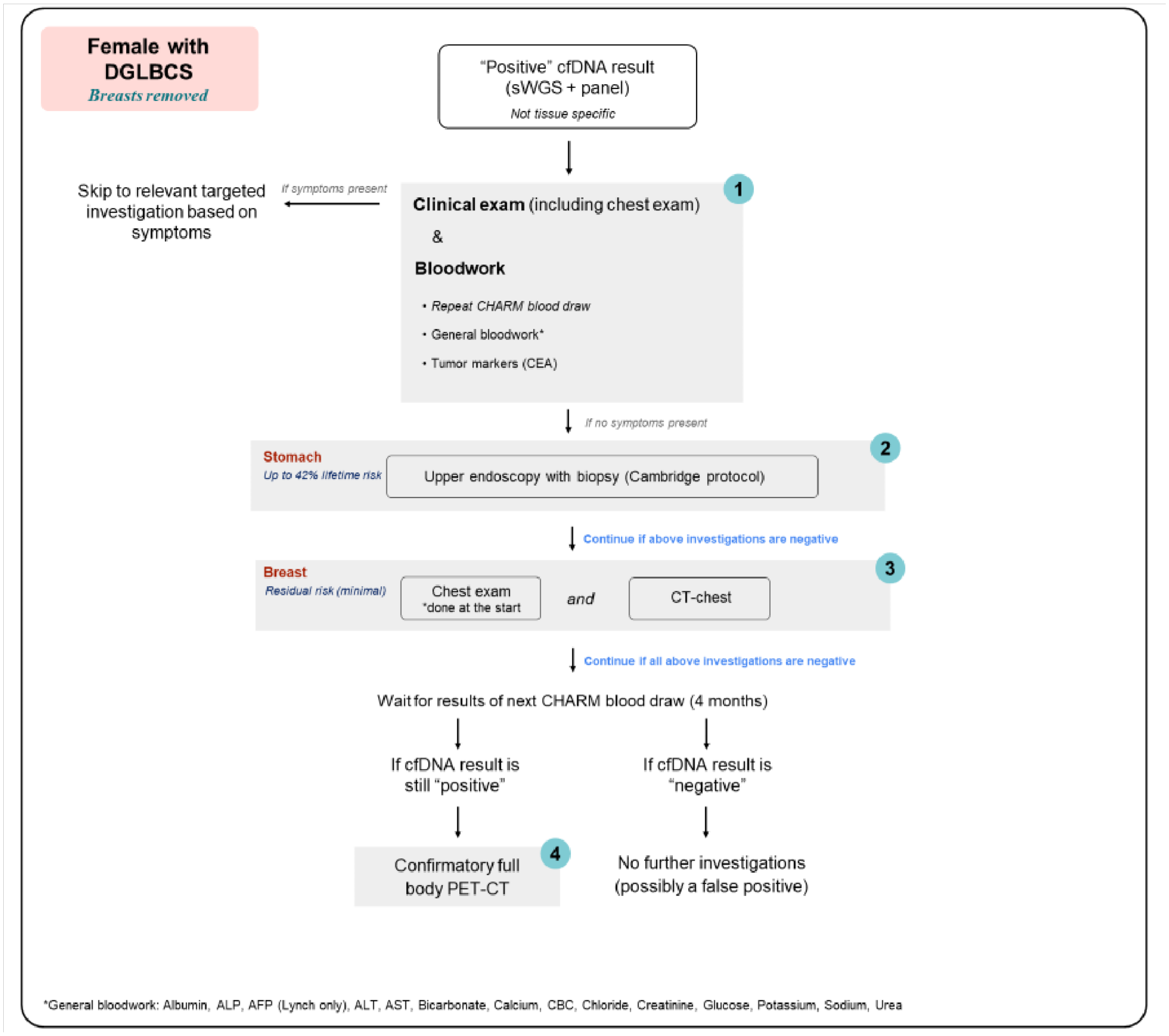

Supplemental Figure S16. Clinical follow-up workflow for Diffuse Gastric and Lobular Breast Cancer syndrome (DGLBCS) cohort for female participants with breasts removed.

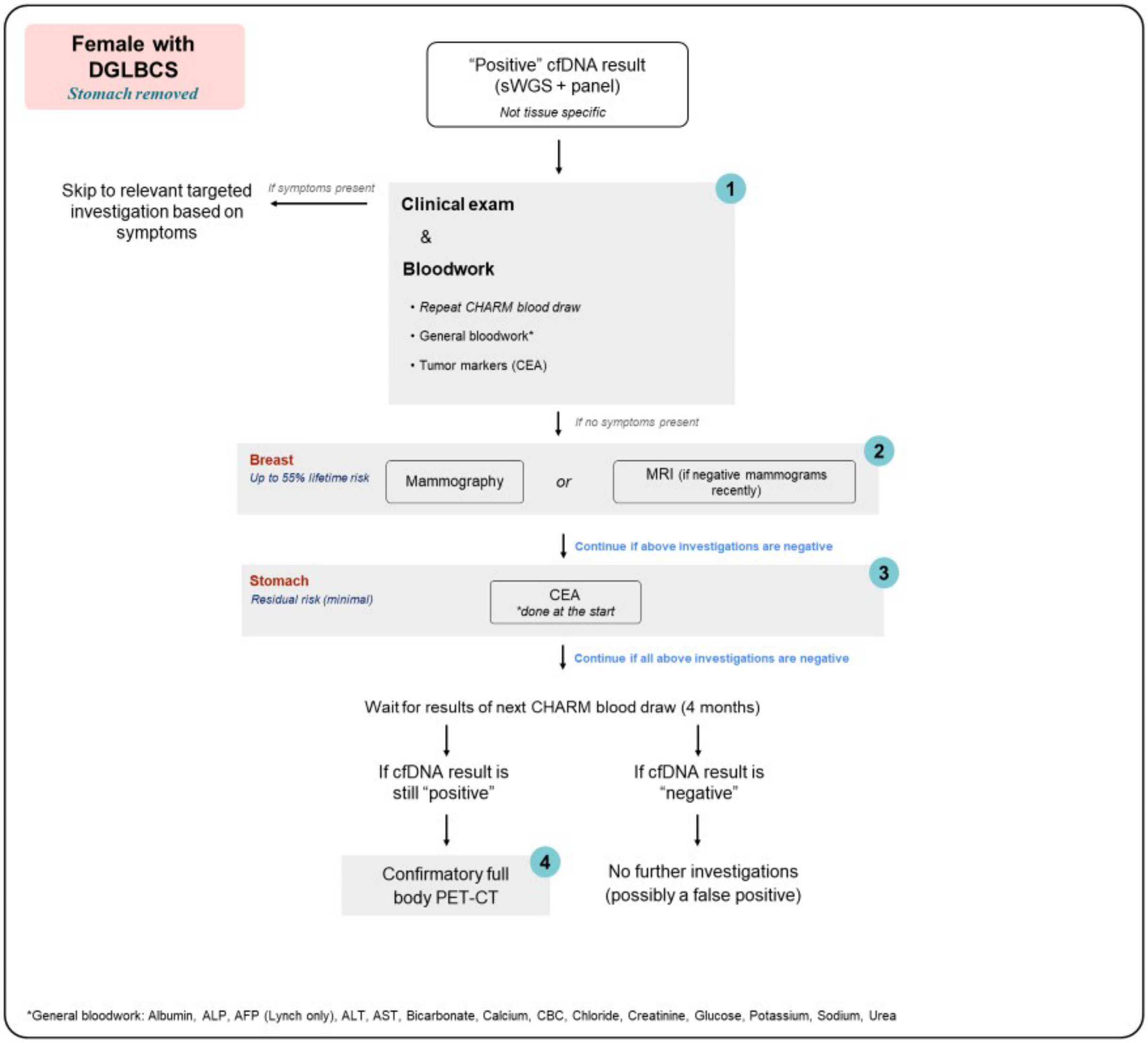

Supplemental Figure S17. Clinical follow-up workflow for Diffuse Gastric and Lobular Breast Cancer syndrome (DGLBCS) cohort for female participants with stomach removed.

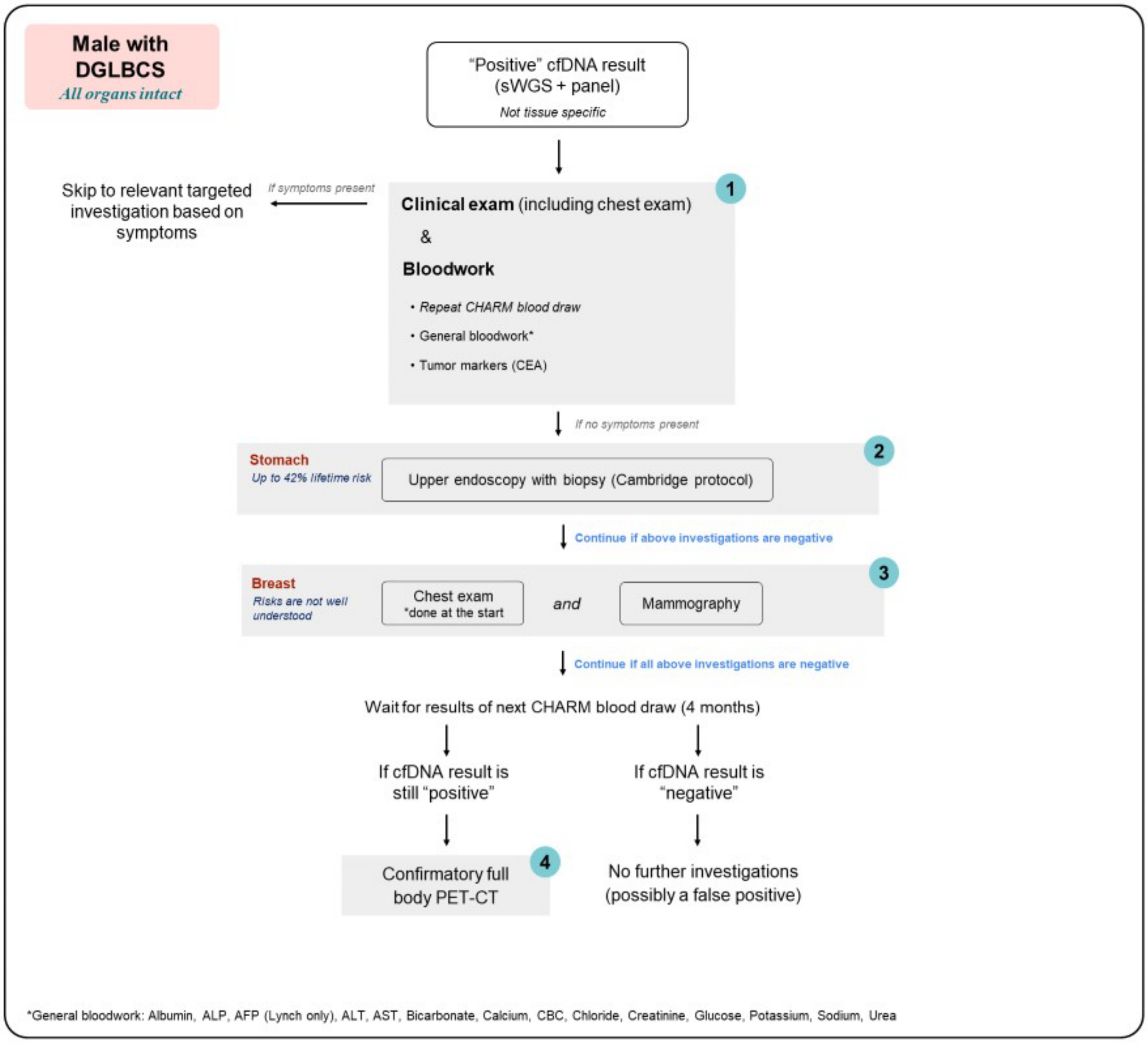

Supplemental Figure S18. Clinical follow-up workflow for Diffuse Gastric and Lobular Breast Cancer syndrome (DGLBCS) cohort for male participants with all organs intact.

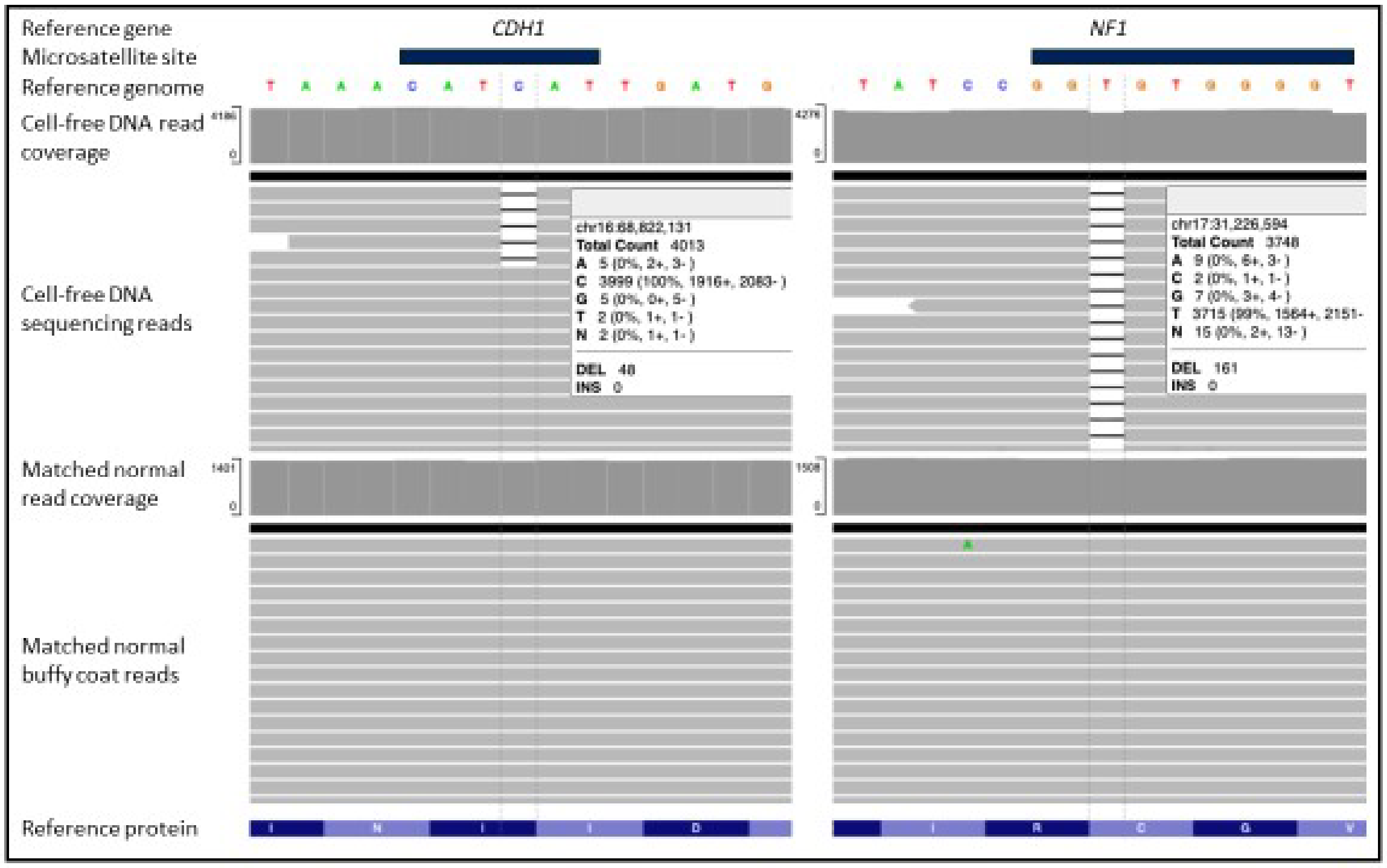

Supplemental Figure S19. Somatic mutations in a microsatellite region within the *NF1* and *CDH1* genes for CHARM2 positive case 2.
